# Inflammatory markers partially mediate the association between volatile organic compounds exposure and hyperlipidemia: a nationally representative cross-sectional study from NHANES

**DOI:** 10.3389/fpubh.2025.1698225

**Published:** 2025-11-26

**Authors:** Yaxiong Nie, Zining He, Bei Liu, Jiaai Li, Yanyu Liu, Xin Su, Zhiqiang Yan, Zheng Li, Chang Yan, Qian Lu, Yanfang Fu, Wanyu Yang, Yutong He

**Affiliations:** 1 Cancer Institute in Hebei Province, The Fourth Hospital of Hebei Medical University, Shijiazhuang, China; 2 School of Public Health, Hebei Medical University, Shijiazhuang, China

**Keywords:** volatile organic compounds, hyperlipidemia, cross-sectional study, inflammatory markers, mediation analysis

## Abstract

**Background:**

While studies have explored associations between volatile organic compounds (VOCs) and metabolic diseases, evidence specifically linking VOCs to hyperlipidemia remains limited. This study aimed to examine the association between urinary VOC metabolites and hyperlipidemia prevalence in U.S. adults.

**Methods:**

Leveraging data from the 2011–2018 National Health and Nutrition Examination Survey (NHANES), this study employed weighted logistic regression, restricted cubic spline (RCS) models, weighted quantile sum (WQS) regression, quantile-based g-computation (qgcomp), and Bayesian kernel machine regression (BKMR) to evaluate associations of individual and mixed VOC exposures with hyperlipidemia. All models were adjusted for covariates including sex, age, race/ethnicity, poverty income ratio (PIR), education level, marital status, body mass index (BMI), smoking status, alcohol consumption, and urinary creatinine. Subgroup analyses assessed effect modifications by sex and age. Multiple-mediator analysis examined the roles of inflammatory biomarkers (white blood cell, lymphocyte, and neutrophil counts) in the association between VOC exposure and hyperlipidemia.

**Results:**

Among 1,979 included participants, weighted logistic regression identified 13 VOC metabolites significantly associated with increased hyperlipidemia risk. Subgroup analyses revealed stronger effects in females and individuals aged <60 years. The RCS model demonstrated positive linear dose–response relationships for hyperlipidemia risk with exposure to xylene, N, N-dimethylformamide, acrylonitrile, crotonaldehyde, 1,3-butadiene, and styrene. Mixture analyses showed significant positive associations via both WQS (1.312 [1.073, 1.606]) and qgcomp (1.296 [1.035, 1.623]), with N, N-dimethylformamide being primary contributor. However, BKMR detected no significant association. Multiple-mediator analysis indicated that inflammatory markers partially mediated the association between the VOC mixture and hyperlipidemia, and WBC was the leading mediator, accounting for 15.094% of the mediated effect.

**Conclusion:**

This study demonstrated significant positive associations of both individual and mixed VOC exposures with hyperlipidemia, with differential susceptibility observed in females and individuals aged <60 years. N, N-dimethylformamide contributed most to the association, and this effect was partly mediated by inflammatory biomarkers.

## 1 Introduction

Hyperlipidemia, a common metabolic disorder characterized by blood lipids, is defined by elevated total cholesterol (TC), low-density lipoprotein cholesterol (LDL-C), and triglycerides (TG), or reduced high-density lipoprotein cholesterol (HDL-C) (1, 2). This condition contributes to insulin resistance and diabetes risk, while promoting arteriosclerosis and stenosis that may lead to hypertension (3). As a major causal factor for cardiovascular disease (CVD)-the leading global cause of death-hyperlipidemia was linked to 610,000 of 2.73 million global ischemic stroke deaths in 2019 (4, 5). Its burden is underscored by high prevalence; in the United States alone, 53% of adults are affected, including 28 million with total cholesterol >240 mg/dL (6.2 mmol/L) (6, 7). Given this significant CVD and diabetes burden, identifying modifiable risk factors is urgent for prevention. In this context, accumulating evidence implicates environmental exposures-notably air pollutants, unhealthy lifestyles, and dietary patterns-in hyperlipidemia pathogenesis (8).

Volatile organic compounds (VOCs), defined as toxic organic chemicals with high vapor pressure at room temperature, are pervasive environmental contaminants originating significantly from industrial emissions (9, 10). Primary human exposure pathways to VOCs include vehicle emissions, household chemicals (cleaning agents, paints), personal care products, and tobacco smoke (11–13). As critical precursors to photochemical oxidants, VOCs participate in heterogeneous atmospheric reactions that generate secondary organic aerosols (SOA) and ozone, key drivers of air quality degradation (14). U.S. monitoring data (2003–2005) revealed persistent exceedances of health benchmarks for key VOCs, including benzene, 1,3-butadiene, and acetaldehyde (15). Substantial experimental and epidemiological evidence demonstrates that VOC exposure induces cardiovascular impairment, chronic respiratory pathologies, and carcinogenesis (16–18). A U.S. cohort study demonstrates that exposure to VOC mixtures elevates risks of all-cause and cause-specific mortality (19). Notably, clinical validation studies confirm that exhaled VOC profiling enables non-invasive early detection of malignancies, particularly lung and gastrointestinal cancers, with VOCs also identified as potential biomarkers for early colorectal cancer screening (20, 21). Consequently, exposure to VOCs is associated with significant health risks.

Humans are exposed to VOCs primarily through inhalation, dietary ingestion, and dermal contact (22). Given their high volatility, VOCs undergo rapid metabolism upon entry into the body. Consequently, they are present at low concentrations in biological fluids (e.g., blood, urine), and their exposure levels can vary considerably due to individual differences and external environmental factors, complicating accurate exposure assessment (23). In contrast, VOC metabolites are inherently more stable, enabling their quantification in blood or urine (24). Due to their enhanced representativeness and stability in urine, urinary VOCs can reflect long-term metabolic alterations, particularly those involving hepatic metabolism. Furthermore, urine collection offers a non-invasive approach for VOC biomonitoring (25, 26). Therefore, urinary VOC metabolites are increasingly used as biomarkers for assessing human exposure to VOCs.

Recent research in public and environmental health has increasingly focused on the association between VOC exposure and adverse population health outcomes (27). Substantial evidence links VOC exposure to an elevated risk of metabolic disorders (28). McGraw et al. found that exposure to VOCs is a relevant but underappreciated environmental factor contributing to cardiovascular disease risk in the general non-smoking population (29). A cohort study conducted in Wuhan, China, identified a significant positive association between specific urinary VOC metabolites during early pregnancy and gestational diabetes (30). A national environmental health survey in South Korea revealed a positive association between urinary VOC metabolites and body mass index (BMI) among Korean adults (31). Despite growing evidence on VOC exposure and metabolic diseases broadly, research specifically examining the association between exposure to complex VOC mixtures and hyperlipidemia risk remains limited. Thus, we conducted a cross-sectional study to examine the association between VOC exposure and hyperlipidemia, and explore its underlying mechanisms.

## 2 Methods

### 2.1 Study population

NHANES is a nationally representative, cross-sectional survey of the US population. It employs a complex, multi-stage probability sampling design to collect sociodemographic characteristics, dietary information, laboratory test results, and questionnaire responses from participants. Detailed information about the NHANES database can be found on its official website.^1^ The study protocol was approved by the National Center for Health Statistics (NCHS)/Centers for Disease Control and Prevention (CDC) Institutional Review Board, and written informed consent was obtained from all participants. This study analyzed data from NHANES 2011–2018, comprising 39,156 participants. After excluding participants with missing data on urinary VOC metabolites, hyperlipidemia-related variables, and key covariates (including sex, age, race, poverty income ratio (PIR), education, marital status, BMI, smoking status, alcohol consumption, urine creatinine), and inflammatory markers, 1,929 participants remained for final analysis (Supplementary Figure S1).

### 2.2 Assessment of hyperlipidemia

Hyperlipidemia was defined per Adult Treatment Panel III (ATP III) guidelines as meeting ≥1 of the following criteria: (1) total cholesterol (TC) ≥ 200 mg/dL; (2) triglycerides (TG) ≥ 150 mg/dL; (3) low-density lipoprotein cholesterol (LDL-C) ≥ 130 mg/dL; or (4) high-density lipoprotein cholesterol (HDL-C) < 50 mg/dL in females or <40 mg/dL in males (32). Additionally, participants using lipid-lowering drugs were defined as having hyperlipidemia.

### 2.3 Measurement of urinary VOCs metabolites

Urine samples (random spot urine samples collected at varying times of the day) were collected, processed, stored, and shipped to the Division of Laboratory Sciences, National Center for Environmental Health, Centers for Disease Control and Prevention, Atlanta GA for analysis. Ultra performance liquid chromatography coupled with electrospray tandem mass spectrometry (UPLC-ESI/MSMS) was used to quantify VOC metabolites in human urine (25). Detailed laboratory methods can be accessed on the NHANES official website (see text footnote 1).

To enhance analytical robustness, VOC metabolites with urinary detection frequencies <80% were excluded. For urine VOC metabolites with concentrations below the limit of detection (LOD), values were imputed as LOD/√2. Finally, Fifteen urinary VOC metabolites were ultimately analyzed: 2-Methylhippuric acid (MHA2), 3-Methylhippuric acid and 4-Methylhippuric acid (MHA3.MHA4), N-Acetyl-S-(2-carbamoylethyl)-L-cysteine (AAMA), N-Acetyl-S-(N-methylcarbamoyl)-L-cysteine (AMCC), 2-Aminothiazol-4-carboxylic acid (ATCA), N-acetyl-S-(benzyl)-L-cysteine (BMA), N-acetyl-S-(2-carboxyethyl)-L-cysteine (CEMA), N-Acetyl-S-(2-cyanoethyl)-L-cysteine (CYMA), N-Acetyl-S-(3,4-dihydroxybutyl)-L-cysteine (DHBMA), N-Acetyl-S-(2-hydroxypropyl)-L-cysteine (HPMA2), N-Acetyl-S-(3-hydroxypropyl)-L-cysteine (HPMA3), N-Acetyl-S-(3-hydroxypropyl-1-methyl)-L-cysteine (HPMMA), N-Acetyl-S-(4-hydroxy-2-buten-1-yl)-L-cysteine (MHBMA3), Phenylglyoxylic acid (PGA), and Mandelic acid (MA). To account for urinary dilution variability, VOC metabolite concentrations were creatinine-adjusted and reported as μg/g creatinine (μg/g Cr).

### 2.4 Measurement of inflammatory markers

Based on the Beckman Coulter methodology of counting and sizing, in combination with an automatic diluting and mixing device for sample processing, and a single beam photometer for hemoglobinometry, blood cell counts were obtained from peripheral blood samples. Our selection of total white blood cell (WBC), lymphocyte, and neutrophil counts as systemic inflammation markers is grounded in prior epidemiological and experimental evidence. These parameters capture complementary facets of innate and adaptive immune activation, have established links to dyslipidemia, and are known to be influenced by VOC exposure. Furthermore, their routine measurement in NHANES makes them practical for large-scale population analyses (33, 34).

### 2.5 Covariates

Key covariates were selected for adjustment via literature synthesis and directed acyclic graph (DAG) construction to minimize potential confounding (35). The adjusted covariates included sex, age, race (Mexican American, Non-Hispanic Black, Non-Hispanic White, Other Hispanic, and Other), PIR, education level, marital status, BMI, smoking status, alcohol consumption, and urinary creatinine level (Supplementary Figure S2). Age was categorized into two groups: <60 years and ≥60 years. PIR represents the ratio of household income to the federal poverty threshold and was classified into three groups: <1.0, 1.0–3.0, >3.0. Education level was categorized into five groups: Less than 9th grade, 9th–11th grade (including 12th grade with no diploma), high school graduate/GED or equivalent, some college or an associate degree (AA), and college graduate or above. Marital status was classified into three groups: Married/Living with a partner, Widowed/Divorced/Separated, and Never married. BMI was calculated as weight (kg) divided by height (m) squared. Smoking status was defined using NHANES items SMQ020 (“Have you smoked at least 100 cigarettes in your lifetime?”) and SMQ040 (“Do you now smoke?”). Participants were classified as never smokers, former smokers, or current smokers. Drinkers were defined as those consuming ≥12 alcoholic drinks annually (36). Urinary metabolite concentrations were expressed as creatinine-standardized values, and urinary creatinine was additionally included as a covariate in multivariable regression models to account for residual variation due to urine dilution (37, 38).

### 2.6 Statistical analysis

Descriptive statistics characterized baseline variables. Continuous data were presented as mean ± standard deviation when normally distributed, or median (interquartile range) otherwise. Group comparisons utilized independent t-tests for normally distributed continuous variables or Wilcoxon rank-sum tests for non-normal distributions. Categorical variables were expressed as frequencies (percentages) with differences assessed by χ^2^ tests. Due to right-skewed distributions of urinary VOC metabolites, natural logarithmic (ln) transformation was performed prior to analysis. Pearson correlation analysis evaluated interrelationships among the 15 urinary VOC metabolites.

Stratification by age (<60 or ≥60 years) and sex (male/female) enabled subgroup-specific analysis of potential differences. Least absolute shrinkage and selection operator (LASSO) regression was applied for variable selection to mitigate multicollinearity. LASSO employs L1 regularization to penalize the regression coefficients, shrinking some to zero for variable selection (39). LASSO inherently “sparsifies” high-dimensional data-it can automatically eliminate noisy variables while preserving true signals and preventing overfitting, thereby improving model interpretability and generalizability (40). Unlike traditional stepwise regression, LASSO does not require manually set selection thresholds, reducing bias from subjective decision-making. The optimal penalty parameter (*λ*) was determined through 10-fold cross-validation with 10,000 repetitions, whereby the final set of selected VOC metabolites was identified based on this optimal value. Based on the VOC metabolites selected by LASSO, we conducted weighted logistic regression using the NHANES sampling weight to evaluate the associations between urinary VOC metabolites and hyperlipidemia, adjusting for relevant covariates. Urinary VOC metabolites were modeled as continuous variables and quartile-based categories, using the first quartile (Q1) as the reference group. Odds ratios (ORs) with 95% confidence intervals (CIs) were estimated per interquartile range (IQR) increase in urinary VOC metabolite concentrations for hyperlipidemia risk. For metabolites categorized into quartiles, tests for linear trend were conducted by modeling the median value of each quartile as a continuous variable in the regression models. To control the cumulative false-positive rate from multiple comparisons, after performing weighted logistic regression on the 13 candidate metabolites, we applied the Benjamini–Hochberg procedure to adjust the raw *p*-values at an FDR ≤ 5%. Metabolites with adjusted *p* < 0.05 were considered statistically significant. The *p*-value from the trend test is used to determine whether the risk of the hyperlipidemia changes linearly with increasing quartiles. Restricted cubic splines (RCS) utilize piecewise polynomial functions to model potential nonlinear relationships between independent and dependent variables (41).

The weighted quantile sum (WQS), quantile-based g-computation (qgcomp), and Bayesian kernel machine regression (BKMR) models were employed to evaluate the association between mixtures of VOCs and hyperlipidemia. For the WQS regression analysis, the data were partitioned into training and testing sets at a ratio of 7:3 (training: testing). To minimize errors arising from random variation, the model was fitted using 1,000 bootstrap resampling iterations. This model stratifies the concentrations of each urinary VOC metabolite into quartiles, assigns differential weights to each quartile, and ultimately generates a weighted composite score termed the WQS index (42). Within this framework, each VOC metabolite is assigned a weight, the value of which delineates its relative contribution to the WQS index. Because WQS regression requires a prespecified uniform direction of exposure–outcome associations, we conducted bidirectional WQS analyses (assuming overall positive and overall negative effects separately) to comprehensively evaluate the potential associations of VOC mixtures. In parallel, we applied the qgcomp method, which does not require a predefined direction and can simultaneously estimate both positive and negative exposure–outcome associations, thereby providing a more flexible assessment of overall effects (43). Additionally, BKMR was employed to assess the association between urinary VOC metabolites and hyperlipidemia. The BKMR model accommodates the complex mixture effects of multiple VOC metabolites and can characterize potential nonlinear relationships and interactions among exposures (44). This approach integrates Bayesian statistics with kernel regression methodology, employing an iterative process to model the exposure-response relationships between VOC metabolites and hyperlipidemia. Using the median concentration of VOC mixtures as the reference, changes in hyperlipidemia risk were examined per decile increment or decrement in mixture concentration to evaluate the association between combined VOC exposure and hyperlipidemia. Moreover, we estimated univariate exposure-response functions and bivariate exposure-response curves for each VOC metabolite to investigate nonlinear effects and interactions among mixture components. All BKMR models were implemented via Markov Chain Monte Carlo (MCMC) with 1,000 iterations.

In addition, a multiple-mediator analysis was conducted to elucidate potential mechanisms linking VOC mixtures with hyperlipidemia. Three inflammatory markers (WBC, lymphocyte, and neutrophil counts) were modeled simultaneously to reflect the immune system’s overall mediating role. Mediation effects were estimated using 1,000 bootstrap resamples, and robustness was examined by repeating the analysis for each mediator separately. To assess the robustness of the association between VOC exposure and hyperlipidemia, we performed three sensitivity analyses. First, we used unadjusted urinary VOC metabolite concentrations as exposure levels. Second, following established practice for biomonitoring quality control, we excluded extreme urine samples (creatinine <30 mg/dL or >300 mg/dL) and repeated our analyses. This standardized creatinine range (30–300 mg/dL) is common in the field and is routinely applied in NHANES-based research and WHO protocols (45). The threshold was not sex-specific because its objective is to flag samples of potential compromised integrity, not to account for individual physiological variation. Finally, because smoking is a major source of VOC exposure, we stratified the sample by self-reported smoking status (never, former, and current smokers) and fitted weighted logistic regression models within each stratum to evaluate the associations between urinary VOC metabolites and hyperlipidemia. All models were adjusted for covariates, including sex, age, race, PIR, education level, marital status, BMI, smoking status, alcohol consumption, and urinary creatinine levels. Visualization were performed using R version 4.4.2, with the packages “survey”, “LASSO”, “gWQS”, “qgcomp”, “plotRCS”, “bkmr”, “Lavaan”, “mediation” and “ggplot2.” Statistical significance was defined as a two-tailed *p*-value < 0.05.

## 3 Results

### 3.1 Baseline demographic characteristics

Table 1 presents the baseline characteristics of the 1,979 participants included in this study from the 2011–2018 NHANES dataset, including 1,023 males (51.693%) and 956 females (48.307%). Among them, 1,181 had hyperlipidemia. Hyperlipidemia was more common in males (*p* < 0.01). Compared with participants without hyperlipidemia, affected individuals had a higher prevalence of obesity (44.200% vs. 29.073%, *p* < 0.001) and increased white blood cell, lymphocyte, and neutrophil counts (all *p* < 0.001). Age, PIR, education, smoking, and alcohol use did not differ between groups, whereas racial distribution did (*p* = 0.039). Specifically, the proportion of Non-Hispanic Black individuals was higher in the non-hyperlipidemia group than in the hyperlipidemia group (23.810% vs. 19.814%), whereas the opposite pattern was observed for Other Hispanic individuals, who showed a higher proportion in the hyperlipidemia group (12.024% vs. 8.396%). The proportions of Mexican American, Non-Hispanic White, and Other Race participants remained relatively stable between groups. Urinary VOC detection rates exceeded 80%; DHBMA and CYMA showed the highest and lowest median concentrations, respectively (345 and 1.73 ng/mL). Supplementary Table S1 presents the names, detection limits, detection rates, and concentration distributions of the 15 urinary VOC metabolites. The VOC metabolites and their corresponding parent compounds are shown in Supplementary Table S2.

**Table 1:** Baseline characteristics of participants among US adults.

Characteristics	Total (*N* = 1979)	Hyperlipidemia		*p* value
		No (*N* = 798)	Yes (*N* = 1,181)	
Sex, *n* (%)				**0.001**
Male	1,023 (51.693)	448 (56.140)	575 (48.688)	
Female	956 (48.307)	350 (43.860)	606 (51.312)	
Age Group, *n* (%)				0.726
<60	1,327 (67.054)	531 (66.541)	796 (67.401)	
≥60	652 (32.946)	267 (33.459)	385 (32.599)	
Race, *n* (%)				**0.039**
Mexican American	254 (12.835)	97 (12.155)	157 (13.294)	
Non-Hispanic Black	424 (21.425)	190 (23.810)	234 (19.814)	
Non-Hispanic White	788 (39.818)	322 (40.351)	466 (39.458)	
Other Hispanic	209 (10.561)	67 (8.396)	142 (12.024)	
Other Race	304 (15.361)	122 (15.288)	182 (15.411)	
PIR				0.323
<1.0	415 (20.970)	164 (20.551)	251 (21.253)	
1.0–3.0	834 (36.887)	324 (40.602)	510 (43.184)	
>3.0	730 (42.142)	310 (38.847)	420 (35.563)	
Education level, n (%)				0.074
Less than 9th grade	152 (7.681)	52 (6.516)	100 (8.467)	
9–11th grade (Includes 12th grade with no diploma)	242 (12.228)	88 (11.028)	154 (13.040)	
High school graduate/GED or equivalent	458 (23.143)	175 (21.930)	283 (23.963)	
Some college or AA degree	609 (30.773)	254 (31.830)	355 (30.059)	
College graduate or above	518 (26.175)	229 (28.697)	289 (24.471)	
Marital Status, n (%)				**<0.001**
Married/Living with partner	1,176 (59.424)	464 (58.145)	712 (60.288)	
Widowed/Divorced/Separated	420 (21.223)	147 (18.421)	273 (23.116)	
Never married	383 (19.35)	187 (23.434)	196 (16.596)	
BMI, n (%)				**<0.001**
Underweight	37 (1.870)	26 (3.258)	11 (0.931)	
Normal	546 (27.590)	274 (34.336)	272 (23.031)	
Overweight	642 (32.441)	266 (33.333)	376 (31.837)	
Obese	754 (38.100)	232 (29.073)	522 (44.200)	
Smoking, *n* (%)				0.136
Current smokers	400(37.071)	144 (18.045)	256 (21.676)	
Former smokers	493(45.690)	207 (25.940)	286 (24.217)	
Never smokers	1,086(54.876)	447 (56.015)	639 (54.107)	
Drinking, *n* (%)				0.576
Yes	1,152 (58.211)	458 (57.393)	694 (58.764)	
No	827 (41.789)	340 (42.607)	487 (41.236)	
Inflammatory markers, (1,000 cells/uL)				
White Blood Cell	6.5 (5.4, 7.9)	6.2 (5.2, 7.4)	6.6 (5.6, 8.1)	**<0.001**
Lymphocyte	1.9 (1.6, 2.4)	1.8 (1.5, 2.3)	2.0 (1.6, 2.4)	**<0.001**
Neutrophils	3.7 (2.9, 4.8)	3.5 (2.7, 4.5)	3.8 (3.0, 4.9)	**<0.001**

Categorical variables are presented as numbers and percentages and compared using the chi-square test, while continuous variables are presented as median (Quartile 1, Quartile 3) and compared using the Wilcoxon test. n, number; PIR, Poverty Income Ratio; BMI, body mass index; Bold, *p* < 0.05.

### 3.2 Association between VOCs and hyperlipidemia

The Pearson correlation analysis showed varying degrees of correlation between the 15 VOC metabolites after ln transformation. Some VOC metabolites exhibited strong correlations (r > 0.7), including MHA2 and MHA3.MHA4 (r = 0.86), CEMA and HPMA3 (r = 0.72), CYMA and HPMMA (r = 0.78), CYMA and MHBMA3 (r = 0.83), HPMA3 and HPMMA (r = 0.83), HPMA3 and MHBMA3 (r = 0.78), HPMMA and MHBMA3 (r = 0.89). Moderate to weak correlations were observed among the remaining VOC metabolites (Supplementary Figure S3).

Using LASSO regression with 10-fold cross-validation (optimal log (*λ*) = −5.941), 13 urinary VOC metabolites associated with hyperlipidemia were selected, including MHA2, MHA3.MHA4, AAMA, AMCC, ATCA, BMA, CEMA, CYMA, HPMA2, HPMMA, MHBMA3, PGA, and MA (Supplementary Figure S4). Table 2 presents the associations between 13 urinary VOC metabolites and hyperlipidemia in the 2011–2018 NHANES dataset. Weighted logistic regression analysis showed that, compared with Q1, the highest quartile (Q4) of the xylene metabolites MHA2 (1.411 [1.003, 1.991]) and MHA3.MHA4 (1.497 [1.070, 1.878]), the N, N-dimethylformamide metabolite AMCC (2.008 [1.361, 2.963]), the cyanide metabolite ATCA (1.570 [1.108, 2.224]), and the acrolein metabolite CEMA (1.495 [1.040, 2.148]) were all significantly associated with an increased risk of hyperlipidemia. Additionally, a positive association was observed between the Q3 of the acrolein metabolite CEMA (1.494 [1.114, 1.953]) and hyperlipidemia risk. Trend tests indicated that the *p*-values for MHA2, MHA3.MHA4, AMCC, and CEMA were all <0.05, suggesting a statistically significant positive linear relationship between the levels of these metabolites and hyperlipidemia risk. In the continuous-variable analysis, adjustment of the raw *p*-values using the Benjamini–Hochberg method did not alter the statistical significance of these metabolites.

**Table 2:** Associations between single urinary VOCs metabolites and hyperlipidemia among US adults.

Variables	Q1	Q2		Q3		Q4		*p* trend	Continuous		adj*_p* value
		OR (95%CI)	*p* value	OR (95%CI)	*p* value	OR (95%CI)	*p* value		OR (95%CI)	*p* value	
MHA2											
Overall	Reference	1.029 (0.734, 1.443)	0.865	1.311 (0.874, 1.968)	0.187	1.411 (1.003, 1.991)	**0.049**	**0.021**	1.131 (1.021, 1.253)	**0.019**	**0.049**
MHA3.MHA4											
Overall	Reference	1.356 (0.940, 1.956)	0.101	1.352 (0.914, 2.001)	0.129	1.497 (1.070, 2.094)	**0.019**	**0.026**	1.139 (1.027, 1.263)	**0.015**	**0.049**
AAMA											
Overall	Reference	1.269 (0.866, 1.861)	0.217	0.764 (0.538, 1.083)	0.128	0.794 (0.557, 1.131)	0.198	0.057	0.828 (0.712, 0.963)	**0.015**	**0.049**
AMCC											
Overall	Reference	1.393 (0.961, 2.020)	0.079	1.329 (0.950, 1.859)	0.095	2.008 (1.361, 2.963)	**0.001**	**0.001**	1.373 (1.164, 1.619)	**<0.001**	**0.003**
ATCA											
Overall	Reference	1.084 (0.736, 1.598)	0.678	0.803 (0.549, 1.173)	0.251	1.570 (1.108, 2.224)	**0.012**	0.065	1.122 (1.000, 1.259)	0.051	0.904
BMA											
Overall	Reference	1.046 (0.739, 1.480)	0.797	1.173 (0.788, 1.746)	0.425	1.080 (0.765, 1.525)	0.657	0.521	1.008 (0.883, 1.151)	0.904	0.904
CEMA											
Overall	Reference	1.329 (0.886, 1.994)	0.166	1.494 (1.144, 1.953)	**0.004**	1.495 (1.040, 2.148)	**0.030**	**0.017**	1.307 (1.119, 1.527)	**0.001**	**0.006**
CYMA											
Overall	Reference	0.990 (0.737, 1.330)	0.947	0.914 (0.653, 1.280)	0.595	1.231 (0.895, 1.693)	0.196	0.320	1.045 (0.986, 1.107)	0.138	0.179
HPMA2											
Overall	Reference	0.984 (0.703, 1.376)	0.922	1.006 (0.673, 1.503)	0.977	0.927 (0.637, 1.349)	0.687	0.743	0.913 (0.789, 1.056)	0.217	0.256
HPMMA											
Overall	Reference	1.092 (0.722, 1.651)	0.672	1.020 (0.764, 1.362)	0.892	1.383 (0.959, 1.993)	0.081	0.122	1.182 (1.017, 1.375)	**0.030**	**0.035**
MHBMA3											
Overall	Reference	0.930 (0.639, 1.354)	0.700	1.034 (0.735, 1.453)	0.847	1.202 (0.833, 1.733)	0.320	0.253	1.120 (0.988, 1.268)	0.075	0.122
PGA											
Overall	Reference	0.828 (0.561, 1.222)	0.335	0.915 (0.638, 1.312)	0.624	1.158 (0.753, 1.780)	0.498	0.418	1.065 (0.903, 1.256)	0.447	0.484
MA											
Overall	Reference	0.922 (0.612, 1.388)	0.692	0.962 (0.647, 1.431)	0.847	1.230 (0.836, 1.810)	0.288	0.296	1.219 (0.972, 1.528)	0.085	0.122

*N* = 1979, The model was adjusted for covariates including sex, age, race/ethnicity, PIR, education level, marital status, BMI, smoking status, alcohol consumption, and urinary creatinine level. Bold, *p* < 0.05.

Sex and age influence the risk of hyperlipidemia (46). Among females, compared to the Q1 group, the Q4 group of the xylene metabolites MHA2 (1.751 [1.156, 2.653]) and MHA3.MHA4 (1.811 [1.127, 2.912]), the N, N-dimethylformamide metabolite AMCC (2.397 [1.448, 3.969]), the cyanide metabolite ATCA (1.516 [1.009, 2.280]), the acrolein metabolite CEMA (1.740 [1.168, 2.591]), the acrylonitrile metabolite CYMA (1.844 [1.135, 2.995]), and the crotonaldehyde metabolite HPMMA (1.476 [1.003, 2.172]) was significantly positively associated with hyperlipidemia risk. The Q3 group of the acrolein metabolite CEMA (1.556 [1.020, 2.347]) and the Q2 group of the N, N-dimethylformamide metabolite AMCC (1.567 [1.004, 2.446]) also showed a significant positive association. Trend tests indicated that the *p*-values for MHA2, MHA3.MHA4, AAMA, AMCC, and CEMA were all <0.05. Among participants aged <60 years, the Q4 group of the xylene metabolite MHA3.MHA4 (1.585 [1.033, 2.430]), the N, N-dimethylformamide metabolite AMCC (2.502 [1.628, 3.844]), and the acrolein metabolite CEMA (1.700 [1.148, 2.518]) was significantly positively associated with hyperlipidemia risk, while Q3 group of the acrolein metabolite CEMA (1.869 [1.254, 2.785]) and the Q2 groups of the N, N-dimethylformamide metabolite AMCC (1.935 [1.307, 2.866]) also exhibited positive associations. Trend tests for AAMA, CEMA, and AMCC were all significant, whereas in the ≥60-year group no statistically significant trends were observed (Supplementary Tables S3, S4).

**Figure 1:**
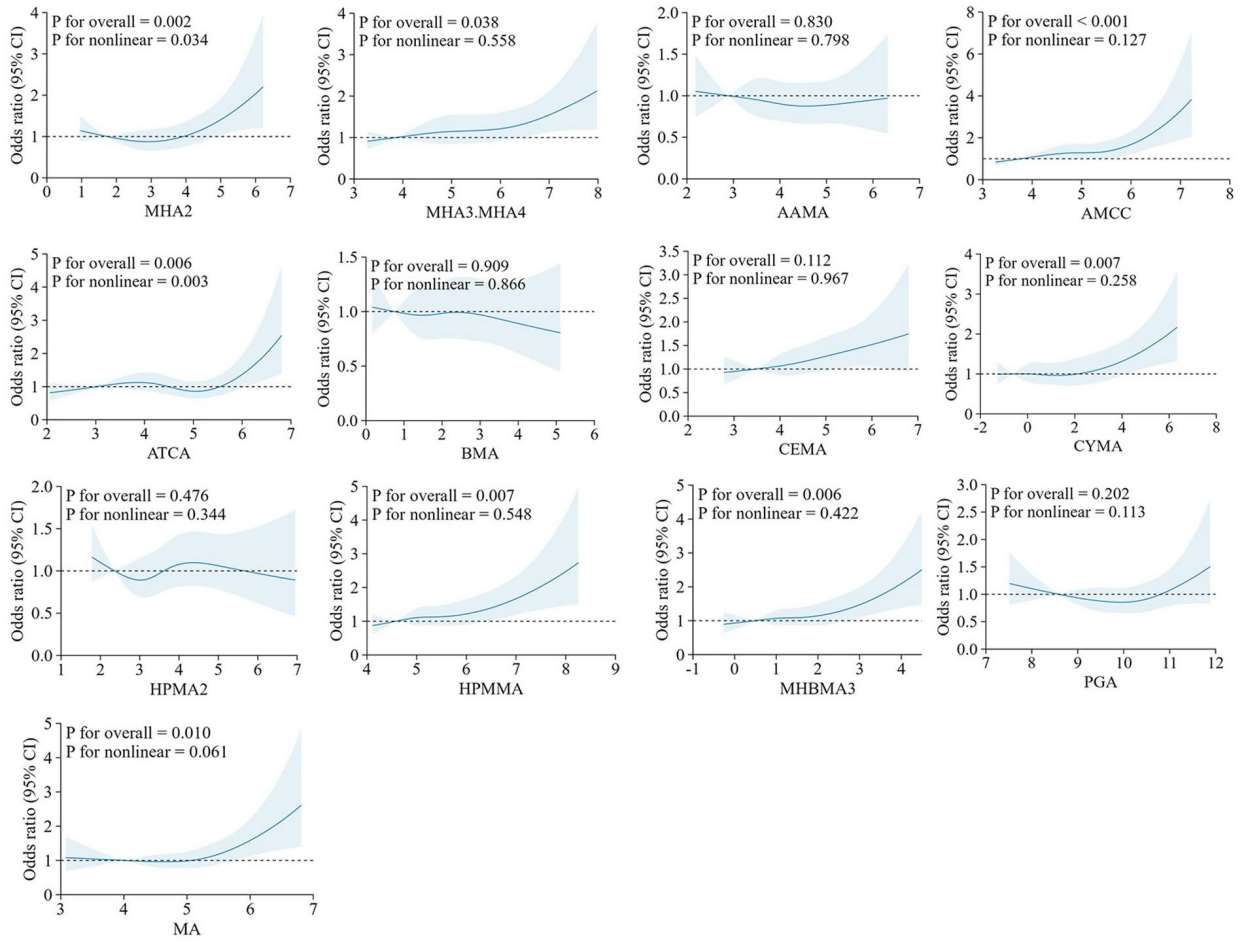
RCS plots of the association between the concentration of urinary VOCs (ln-transformed) and hyperlipidemia. The model was adjusted for covariates including sex, age, race/ethnicity, PIR, education level, marital status, BMI, smoking status, alcohol consumption, and urinary creatinine level. RCS, Restricted Cubic Splines; VOCs, volatile organic compounds; PIR, poverty-income ratio; BMI, body mass index.

RCS analysis of the LASSO-selected VOC metabolites showed that in the overall population, MHA3.MHA4, AMCC, CYMA, HPMMA, MHBMA3, and MA were positively linearly associated with hyperlipidemia risk, whereas MHA2 and ATCA exhibited nonlinear relationships (MHA2 followed a “U” shape and ATCA an “S” shape) (Figure 1). In females, CEMA, HPMMA, and MHBMA3 showed linear dose–response relationships, while the other metabolites exhibited nonlinear patterns; in males, only ATCA was nonlinear (Supplementary Figure S5). Among participants aged <60 years, MHA2, MHA3.MHA4, AMCC, CEMA, HPMMA, and MHBMA3 displayed linear dose–response relationships, whereas ATCA was nonlinear. No significant dose–response relationships were observed in participants aged ≥60 years (Supplementary Figure S6).

### 3.3 Mixture co-exposure analysis

**Figure 2:**
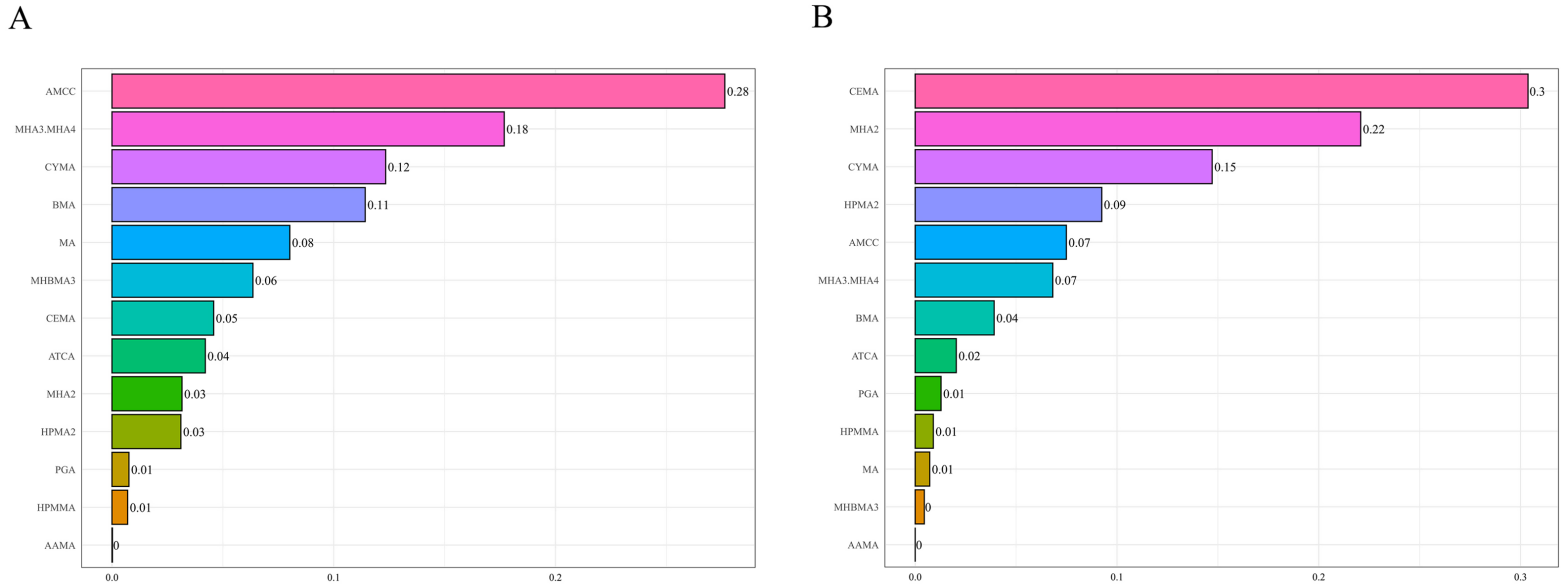
WQS model results for the association between VOCs mixtures (ln-transformed) and hyperlipidemia. Panels display the relative contribution (weights) of each metabolite under two directional assumptions: **(A)** positive-direction WQS model and **(B)** negative-direction WQS model. The model was adjusted for covariates including sex, age, race/ethnicity, PIR, education level, marital status, BMI, smoking status, alcohol consumption, and urinary creatinine level. WQS, weighted quantile sum; VOCs, volatile organic compounds.

We applied WQS regression to VOC metabolites selected by LASSO under both positive- and negative-direction assumptions to evaluate their association with hyperlipidemia. In the positive-direction WQS model, the WQS index was significantly positively associated with hyperlipidemia in the overall sample (1.312 [1.073, 1.606]), with AMCC carrying the largest weight (0.280) (Figure 2). This association remained significant among females and participants aged <60 years. In the <60-year group, MHA3.MHA4 had the highest weight (0.350), while AMCC had the largest weight in females (0.270). In the negative-direction WQS model, the WQS index was significantly inversely associated with hyperlipidemia (0.816 [0.677, 0.984]), suggesting a potential protective effect of the mixture, with CEMA (0.300) and MHA2 (0.220) contributing most. In females and the <60-year group, MHA2 carried the highest weight (Supplementary Figure S7 and Supplementary Table S5).

**Figure 3:**
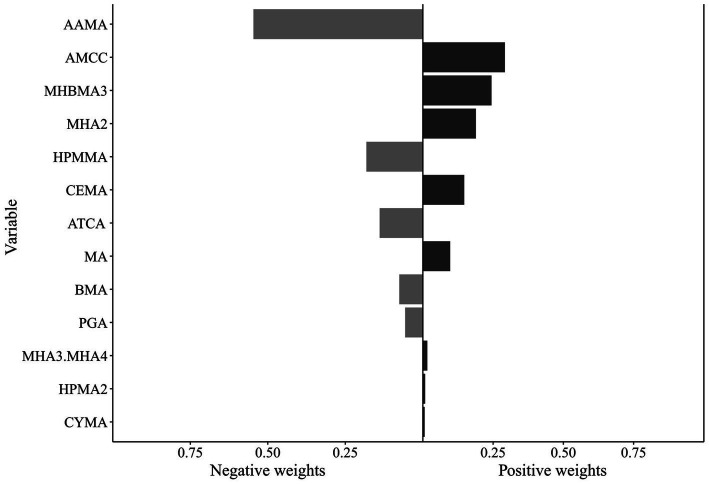
Qgcomp model results for the association between VOCs mixtures (ln-transformed) and hyperlipidemia. The model was adjusted for covariates including sex, age, race/ethnicity, PIR, education level, marital status, BMI, smoking status, alcohol consumption, and urinary creatinine level. Qgcomp, Quantile-based g computation method; VOCs, volatile organic compounds.

Qgcomp analysis revealed a significant positive association between the VOC mixture and hyperlipidemia risk (1.296 [1.035, 1.623]) (Supplementary Table S6). Qgcomp analysis calculated positive and negative weights for each VOC metabolite. Metabolites with positive weights included AMCC (0.292), MHBMA3 (0.244), MHA2 (0.189), CEMA (0.147), MA (0.097), MHA3 and MHA4 (0.016), HPMA2 (0.008), and CYMA (0.006), among which AMCC exhibited the highest positive weight. Metabolites with negative weights were AAMA (0.546), HPMMA (0.182), ATCA (0.139), BMA (0.076), and PGA (0.057), with AAMA demonstrating the largest negative weight (Figure 3). Stratified analysis results were consistent with the WQS model, revealing a significant positive association between the VOC mixture and hyperlipidemia risk among females (1.460 [1.123, 1.897]) and individuals aged <60 years (1.296 [1.035, 1.623]). However, among males (0.980 [0.769, 1.249]) and individuals aged ≥60 years (0.998 [0.726, 1.371]), the association between the VOC mixture and hyperlipidemia risk was inverse but not statistically significant. Among females, CEMA (0.231), MHBMA3 (0.228), MHA2 (0.189), AMCC (0.148), PGA (0.122), ATCA (0.041), HPMA2 (0.023), and CYMA (0.018) exhibited positive weights, with CEMA exhibiting the highest. In the <60 years age group, AMCC had the largest positive weight (0.239), mirroring the result observed in the overall population. In both the female subgroup and the <60 years age group, AAMA exhibited the largest negative weights (0.670 and 0.569, respectively) (Supplementary Figure S8).

**Figure 4:**
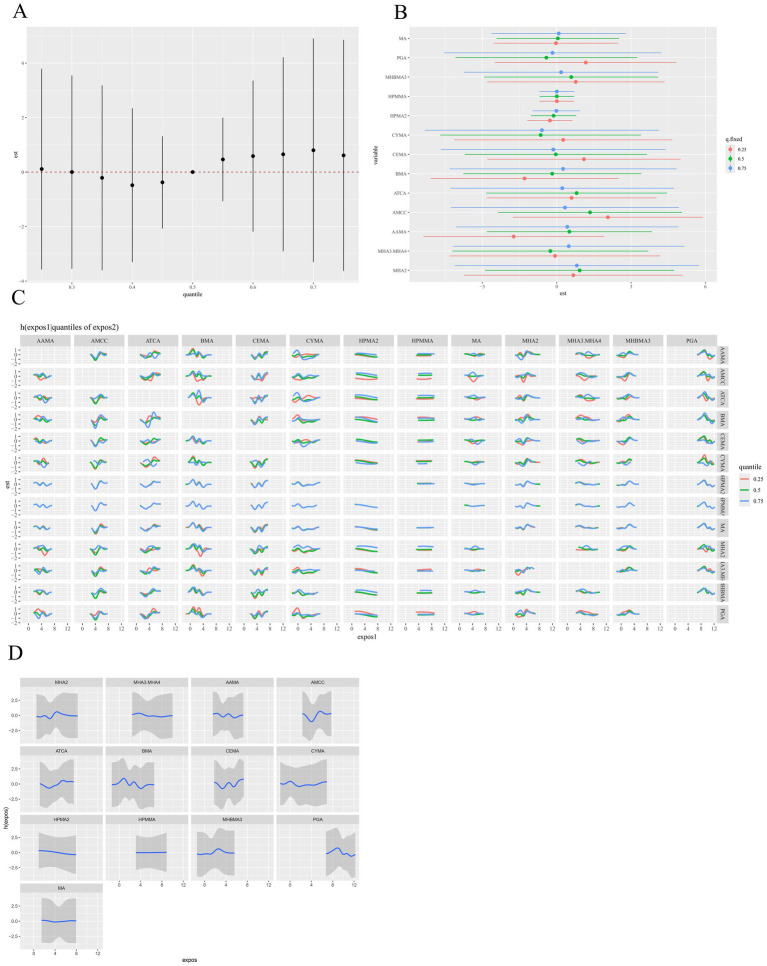
BKMR model results for the association between VOCs mixtures (ln-transformed) and hyperlipidemia. **(A)** The overall relationship between VOCs mixtures and hyperlipidemia, by comparing the value of h when all of VOCs are at a particular percentile as compared to when all of them are at their 50th percentile. **(B)** The single-exposure risk of each VOC on hyperlipidemia when other VOCs were fixed at the 25th, 50th, or 75th percentile. **(C)** Response of a single VOC for the second VOC fixed at various percentiles (25th, 50th, 75th), and for the remaining VOCs fixed at the 50th percentile. **(D)** The univariate dose–response relationship between each VOC and hyperlipidemia when all other VOCs were fixed at the 50th percentile. The model was adjusted for covariates including sex, age, race/ethnicity, PIR, education level, marital status, BMI, smoking status, alcohol consumption, and urinary creatinine level. BKMR, Bayesian kernel machine regression; VOCs, volatile organic compounds.

The MCMC sampling for the BKMR models displayed good convergence. The overall Metropolis–Hastings acceptance rate was 0.451, and inspection of trace plots for the covariate regression coefficients showed stable behavior across iterations (Supplementary Figure S9). The BKMR model revealed a significant positive overall association between the VOC mixture and hyperlipidemia risk. Specifically, elevated concentrations of the mixture were associated with progressively higher risks compared to the 50th percentile (Figure 4A). In further investigating the relationship between individual VOC metabolites and hyperlipidemia, we fixed the concentrations of all other VOC metabolites at median levels and estimated the corresponding univariate exposure-response function for each metabolite (Figure 4D). The results demonstrated that AAMA, BMA, and PGA exhibited inverse, nonlinear associations with hyperlipidemia, whereas AMCC and ATCA showed positive and nonlinear associations. No clear trends were observed for the remaining metabolites. To further explore potential interactions among VOC metabolites, we quantified the impact of individual metabolites on hyperlipidemia while holding the concentrations of others constant at their 25th, 50th, and 75th percentiles (Figure 4B). AMCC was positively associated with hyperlipidemia. When the remaining VOC metabolites were fixed at their 25th and 50th percentiles, the point estimates for AMCC were largest. However, the corresponding 95% posterior credible bands included the null value, so these associations did not reach statistical significance within the BKMR framework. Bivariate exposure-response function analysis suggested potential interactions among other VOC metabolites, with the exception of HPMA2 and HPMMA (Figure 4C). Furthermore, subgroup analyses stratified by gender and age were performed to investigate the association between VOC metabolite mixtures and hyperlipidemia across these subgroups. However, these analyses revealed no significant differences across gender or age groups (Supplementary Figures S10–S13).

### 3.4 Mediation analysis

**Figure 5:**
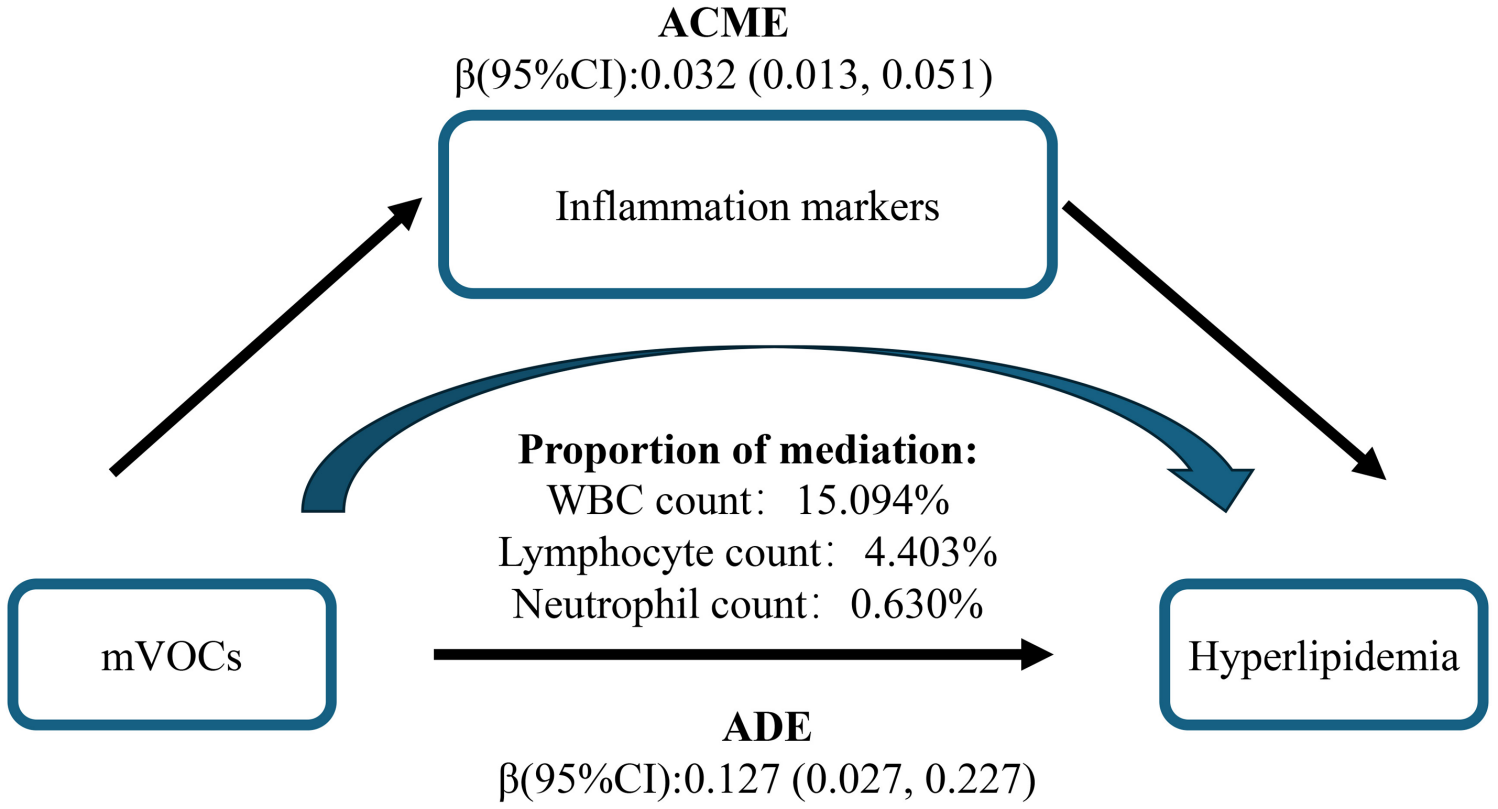
Mediation effects of multiple inflammatory markers in the association between urinary VOCs and hyperlipidemia. The model was adjusted for covariates including sex, age, race/ethnicity, PIR, education level, marital status, BMI, smoking status, alcohol consumption, and urinary creatinine level. VOCs, volatile organic compounds; ACME, Average Causal Mediation Effect; ADE, Average Direct Effect.

In the multiple-mediator model, the overall mediating effect of inflammatory markers was significant. WBC was the primary mediator, accounting for 15.094% of the mediation, whereas the indirect effects of lymphocytes and neutrophils were small and not statistically significant, with mediation proportions of 4.403 and 0.630%, respectively (Figure 5 and Supplementary Table S7). To assess the robustness of the results, we entered each of the three immune cells separately into single-mediator models. All three showed significant mediating effects, with WBC again accounting for the largest proportion, indicating that WBC plays a stable mediating role in the pathway from VOC mixture exposure to hyperlipidemia. This supports the robustness of the conclusions from the multiple-mediator model (Supplementary Figure S14). As single-mediator analyses often yield larger indirect effects and mediation proportions (Supplementary Table S8), the multiple-mediator model was adopted as the primary evidence in this study to avoid overestimation of individual mediation effects.

### 3.5 Sensitivity analysis

To assess the robustness of the association between VOC metabolite exposure and hyperlipidemia, we performed three sensitivity analyses. First, urinary VOC metabolite concentrations uncorrected for creatinine were used as the exposure metric. The results demonstrated that the association between VOC metabolite exposure and hyperlipidemia remained consistent with the main analysis. Second, samples with urinary creatinine concentrations below 30 mg/dL (indicating dilution) or above 300 mg/dL (indicating concentration) were excluded, and the association analysis was repeated. After excluding these samples, the association between VOC metabolite exposure and hyperlipidemia remained consistent with the main findings. Finally, stratified sensitivity analyses were conducted according to smoking status (never, former, and current smokers). After Benjamini–Hochberg correction, the metabolites MHA2, MHA3.MHA4, AAMA, AMCC, and CEMA were all significantly positively associated with hyperlipidemia. These results were consistent with the analyses in the overall population and thereby strengthen the robustness of the primary findings. Notably, the significant associations were mainly observed among current smokers, suggesting that smoking may represent an important source of exposure to these VOCs and that smoking-related exposures may be linked to hyperlipidemia. In conclusion, the sensitivity analyses support the robustness of the observed association between VOC metabolite exposure and hyperlipidemia across varying analytical approaches, underscoring the reliability of the study’s conclusions (Supplementary Tables S9–S11).

## 4 Discussion

This study employed a multi-model approach to systematically investigate the association between VOC metabolite exposure and hyperlipidemia and explore potential underlying mechanisms. This study examined 12 VOCs: xylene (MHA2, MHA3.MHA4), acrylamide (AAMA), N, N-dimethylformamide (AMCC), cyanide (ATCA), toluene (BMA), acrolein (CEMA, HPMA3), acrylonitrile (CYMA), 1,3-butadiene (DHBMA, MHBMA3), propylene oxide (HPMA2), crotonaldehyde (HPMMA), ethylbenzene (PGA), and styrene (PGA, MA). Multivariable weight logistic regression identified significant positive associations between hyperlipidemia risk and exposure to xylene, acrylamide, N, N-dimethylformamide, acrolein, crotonaldehyde, with stronger effects observed in females and individuals < 60 years. RCS models further revealed positive linear dose–response relationships for xylene, N, N-dimethylformamide, acrylonitrile, crotonaldehyde, 1,3-butadiene, and styrene. In mixture exposure analyses, WQS and qgcomp models indicated N, N-dimethylformamide as the primary contributors to hyperlipidemia risk. While BKMR did not detect a significant overall mixture effect, it suggested a potential positive trend. Additionally, bivariate analyses suggested potential interactions among other VOC metabolites, excluding propylene oxide and crotonaldehyde. To our knowledge, this is the first study to comprehensively evaluate the VOC metabolite-hyperlipidemia relationship, providing detailed evidence for environmental health research.

The progression of industrialization and urbanization, coupled with persistent fossil fuel combustion, has exacerbated the global burden of air pollution, establishing it as the predominant environmental risk factor for human disease and premature mortality (47, 48). Substantial epidemiological evidence demonstrates a robust association between air pollution and the risk of cardiovascular diseases (49–51). A prospective study of older adults in the United States indicated that elevated cholesterol levels partially mediate the association between air pollution and cognitive decline (52). Three cross-sectional studies conducted in the United States (53), Taiwan (54), and China (55), along with a retrospective cohort study in Israel (56), have all demonstrated that long-term exposure to air pollution is associated with elevated LDL-C levels, thereby providing further support for the link between air pollution and hyperlipidemia. A study conducted in Taiwan linked residential proximity to petrochemical industrial zones and exposure to elevated mercury and arsenic levels with an increased risk of dyslipidemia, including hyperlipidemia (57). Notably, research involving 12,000 Native Americans demonstrated that long-term exposure to persistent organic pollutants (polychlorinated biphenyls and organochlorine pesticides) significantly elevates hyperlipidemia risk (58). While current research predominantly focuses on outdoor air pollution health impacts, indoor pollution may pose a greater health threat due to increased building envelope tightness, inadequate ventilation, and prolonged occupancy durations (12). VOCs, ubiquitous in household products, are prevalent indoor pollutants. Notably, U.S. Environmental Protection Agency (USEPA) data indicate that indoor VOC concentrations are typically 2- to 5-fold higher than outdoor levels (59). Therefore, a comprehensive assessment of the association between VOC exposure and hyperlipidemia is of significant public health importance and provides novel scientific insights for environmental health sciences.

Current evidence directly linking VOC exposure to hyperlipidemia is scarce. Existing research, primarily focusing on the effects of VOC exposure on lipid profiles, remains limited. For instance, epidemiological studies indicate that among females, each unit increase in log-transformed blood acrylamide levels is associated with a 2.83 mg/dL increase in TC levels (60). Experimental evidence indicates that acrylamide binding to lipoproteins can impair HDL function and reduce its cholesterol clearance capacity, thereby elevating hyperlipidemia risk (61). Supporting these findings, animal studies demonstrate that 21-day exposure to aluminum and acrylamide significantly increased plasma TC and LDL-C levels, while decreasing HDL-C and TG levels in rats (62). Furthermore, a cohort study in Taiwan identified a positive correlation between 1,3-butadiene exposure and LDL-C levels (63). Subgroup analyses further indicated that females and individuals aged <60 years are significantly more susceptible to VOC exposure effects than males and those aged ≥60 years. The greater susceptibility of females to VOC exposure may be partly attributable to their higher average body fat percentage. Many VOCs are lipophilic and can accumulate in adipose tissue, potentially resulting in higher chronic exposure levels (64, 65). Second, estrogen can potentiate VOC-induced oxidative stress responses, which may subsequently disrupt lipid metabolism (66). Moreover, females generally exhibit higher usage frequencies of personal care products (e.g., perfumes, nail polish) and undertake more household cleaning activities, resulting in exposure to diverse sources of household chemicals (67). The heightened susceptibility of individuals aged <60 years to VOC-associated hyperlipidemia may stem from their higher metabolic rate, potentially accelerating VOC absorption and metabolic processing (68).

In stratified sensitivity analyses by smoking status, the positive association between VOCs and hyperlipidemia was largely confined to current smokers. Smoking substantially increases internal VOC burden (47, 48), and St. Helen et al. reported that urinary VOC metabolite concentrations were 1.31–7.09 times higher in smokers than in non-smokers (69). Thus, the association between VOCs and hyperlipidemia observed among smokers may partly reflect short-term, elevated VOC exposure resulting from smoking. Numerous epidemiological studies have shown that smoking is associated with hyperlipidemia, particularly increases in total cholesterol, LDL-C, and triglycerides, potentially mediated by oxidative stress and chronic inflammation induced by smoking (70–72). Taken together, smoking status may function both as a source of VOC exposure and as a confounding factor in the observed association between VOCs and hyperlipidemia. Given the cross-sectional design of our study, causal inference is limited; longitudinal cohort studies and mechanistic analyses are warranted to further disentangle these complex relationships.

While previous research has predominantly examined the effects of individual VOCs on lipid profiles, real-world exposure typically involves complex VOC mixtures. Single-compound analysis may therefore inadequately represent actual exposure scenarios and their health risks. Epidemiological evidence supports this concern; notably, a study of nail salon workers chronically exposed to low-level VOC mixtures reported significantly elevated plasma TG levels and TC to HDL-C (TC/HDL-C) ratios, alongside significantly reduced HDL-C levels (73). Furthermore, a study of petroleum industry workers in Iran found that chronic exposure to VOC mixtures was associated with significantly elevated plasma levels of TG, TC, and LDL-C (74). However, findings from these studies in distinct occupational settings may have limited generalizability to the general population. To address this gap, we applied WQS regression, qgcomp, and BKMR models to assess the association between mixed VOC exposure and hyperlipidemia in a nationally representative sample of U.S. adults. Both WQS and qgcomp analyses consistently indicated a significant positive association between the VOC mixture and hyperlipidemia risk. While the BKMR model did not identify a statistically significant association, it indicated a positive trend towards an association. Both the WQS and qgcomp models identified N, N-dimethylformamide (DMF) as the largest contributor to hyperlipidemia, which may be related to its hepatotoxicity (75, 76). The liver plays a pivotal role in lipid metabolism. Fatty acids, transported bound to albumin in the bloodstream, enter hepatocytes through specific transport proteins. Within hepatocytes, fatty acids can undergo *β*-oxidation to generate energy or be esterified into TG for storage. Subsequently, TG is packaged into very low-density lipoprotein (VLDL) particles and secreted into the circulation, thereby contributing to systemic lipid metabolism (77). Consequently, liver injury can result in hyperlipidemia by disrupting lipid metabolism. Collectively, our findings elucidate the potential role of mixed VOC exposure in the development of hyperlipidemia and provide novel empirical evidence supporting epidemiological research in this field.

A previous cross-sectional study established a significant positive correlation between inflammatory markers and hyperlipidemia (78). In the multiple-mediator model, the overall mediating effect of inflammatory markers was significant. WBC was the primary mediator, accounting for 15.094% of the mediation. This is consistent with established knowledge that chronic environmental VOC exposure can trigger oxidative stress and inflammatory responses (79, 80). Upon entering the body, VOCs are metabolically converted to generate reactive oxygen species (ROS), eliciting oxidative stress, which subsequently activates signaling pathways, promotes inflammatory mediator release, and ultimately leads to systemic inflammation (81). Specifically, xylene can impair the cellular antioxidant defense system, elevating ROS levels and thereby inducing oxidative stress and damage (82). Similarly, styrene elicits inflammatory responses by compromising cellular antioxidant capacity and activating inflammatory signaling pathways (83). Additionally, a study reported that subjects relocating to new buildings characterized by higher VOC levels showed significantly elevated urinary leukotriene E4 levels, indicating enhanced non-Th2 inflammatory responses (84). Another animal study demonstrated that VOC exposure (N-methyl-2-pyrrolidone [NMP] and 2,2,4-trimethyl-1,3-pentanediol diisobutyrate [TXIB]) from PVC flooring induced oxidative stress, dendritic cell dysfunction, elevated Th2 cytokine levels, and eosinophilic pulmonary inflammation in mice (85). Collectively, these studies provide evidence supporting the pro-inflammatory effects of VOCs. Notably, the role of inflammation in hyperlipidemia development warrants consideration. A cross-sectional study identified an inverted U-shaped relationship between the systemic immune-inflammation index (SII) and hyperlipidemia, with an inflection point at 479.15. Below this threshold, SII served as an independent risk factor for hyperlipidemia (86). Furthermore, patients with inflammatory bowel disease (IBD), including Crohn’s disease and ulcerative colitis, exhibit significantly elevated circulating inflammatory cytokine levels, frequently accompanied by reduced HDL-C and elevated LDL-C levels (87). Complementing this, a metabolomics study revealed a similar mechanism, demonstrating that polysaccharides can alleviate hyperlipidemia by attenuating oxidative stress and suppressing inflammatory factor expression (88). In summary, the impact of VOC exposure on hyperlipidemia entails complex mechanisms, wherein inflammation plays a significant role. Further research is warranted to delineate the mechanisms by which VOCs affect lipid metabolism through inflammation and other molecular pathways.

This study has several strengths. Primarily, it represents the first comprehensive investigation into the association between individual and mixed VOCs exposure and hyperlipidemia in a nationally representative sample of U.S. adults, establishing novel epidemiological evidence for this field. Secondarily, our mediation analysis identified inflammatory markers as potential mediators in the relationship between VOCs exposure and hyperlipidemia, offering new insights into the underlying biological mechanism. Finally, sensitivity analyses were performed to verify the robustness of our primary findings, thereby strengthening the reliability of the study. Despite these strengths, this study has several limitations. First, the cross-sectional design precludes causal inference regarding the association between VOC exposure and hyperlipidemia. Second, although numerous covariates were adjusted for in the analyses to minimize confounding, bias from unmeasured or unknown confounders cannot be completely ruled out. Additionally, VOC exposure levels, which are subject to multiple influences (environmental, occupational, lifestyle) and temporal fluctuations, may lead to potential exposure misclassification. This study assessed VOC exposure using single spot urine specimens, which primarily reflect recent exposure and may not represent chronic exposure levels at the individual level. Future studies should incorporate multiple urine collections or 24-h urine sampling to obtain more robust exposure estimates and attenuate exposure misclassification. Moreover, owing to differences in matrix and exposure time windows, as well as inconsistencies in sample sources and analytical methods, we did not perform a direct comparison between our results and the NHANES serum VOC measurements. To improve comparability and interpretability, future work should undertake cross-matrix comparisons when paired blood–urine samples or harmonized analytical platforms become available. Finally, while mediation analysis provided preliminary evidence for potential mechanisms linking VOC exposure to hyperlipidemia, the biological plausibility of these pathways requires further validation. Our mediation findings are interpreted as supporting a biologically plausible hypothesis, suggesting that inflammation may be a plausible pathway linking VOC exposure to abnormal lipid metabolism. This does not, however, rule out reverse causation or bidirectional effects. Future research should integrate experimental models with longitudinal cohort studies to elucidate the mechanisms underlying VOC-induced hyperlipidemia development and progression.

## 5 Conclusion

In summary, we observed a significant positive association between both individual and mixed VOC exposures and hyperlipidemia. Notably, females and individuals aged < 60 years showed greater susceptibility to this association. N, N-dimethylformamide was identified as the primary contributor to hyperlipidemia risk within mixed VOC exposures. Multiple-mediator analysis revealed that inflammatory markers play a significant mediating role in the relationship between VOC exposure and hyperlipidemia.

## Data Availability

Publicly available datasets were analyzed in this study. This data can be found here: https://wwwn.cdc.gov/nchs/nhanes/Default.aspx.

## Footnotes

1
https://www.cdc.gov/nchs/nhanes
