# Supplementary material for: Inflammatory markers partially mediate the association between volatile organic compounds exposure and hyperlipidemia: a nationally representative cross-sectional study from NHANES
Source: Front Public Health. 2025 Nov 26;13:1698225. doi: 10.3389/fpubh.2025.1698225 (PMC12689905; doi:10.3389/fpubh.2025.1698225)
Supplement: Supplementary file 1 [file Data_Sheet_1.docx]

**Supplementary Material**

**Inflammatory markers partially mediate the association between volatile organic compound exposure and hyperlipidemia: A nationally representative cross-sectional study from NHANES**

Yaxiong Nie^a,b^, Zining He^a,b^, Bei Liu^a,b^, Jiaai Li^a,b^, Yanyu Liu^a,b^, Xin Su^a,b^, Zhiqiang Yan^a,b^, Zheng Li^a,b^, Chang Yan^a,b^, Qian Lu^a,b^, Yanfang Fu^a,b^, Wanyu Yang^a,b^, Yutong He^a*^

^a^Cancer Institute in Hebei Province, The Fourth Hospital of Hebei Medical University, Shijiazhuang, China

^b^School of Public Health, Hebei Medical University, Shijiazhuang, China

*Correspondence: Yutong He, PhD, Cancer Institute in Hebei Province, The Fourth Hospital of Hebei Medical University, School of Public Health, Hebei Medical University, 12 Jiankang Road, Shijiiazhuang, China, 050000, E-mail: heyutong@hebmu.edu.cn. Tel: 0311-86095613

**This file includes:**

**Table S1.** Distribution of urinary VOC metabolites (ng/mL) among US adults.

**Table S2** VOC metabolites and their parent compounds.

**Table S3.** Sex-stratified associations between individual urinary VOCs and hyperlipidemia among US adults.

**Table S4.** Age-stratified associations between individual urinary VOCs and hyperlipidemia among US adults.

**Table S5.** Associations between the WQS regression index and hyperlipidemia among US adults.

**Table S6.** Associations between urinary VOCs and hyperlipidemia among US adults: A quantile g-computation approach.

**Table S7.** Inflammation markers as multiple mediators in the association between VOCs mixture and hyperlipidemia among US adults.

**Table S8.** Inflammation markers as single mediators in the association between VOCs mixture and hyperlipidemia among US adults.

**Table S9.** Sensitivity analysis of the association between VOCs and hyperlipidemia without urinary creatinine adjustment.

**Table S10.** Sensitivity analysis of the relationship between urinary VOCs and hyperlipidemia after excluding individuals with urinary creatinine >300 mg/L or urinary creatinine <30 mg/L.

**Table S11.** Sensitivity analysis of the association between VOC and hyperlipidemia based on different smoking statuses.

**Figure S1.** Flowchart for the selection of participants. NHANES, the National Health and Examination Survey; VOCs, volatile organic compound.

**Figure S2.** Covariate selection for evaluating the impact of urinary VOCs on hyperlipidemia was guided by a DAG.

**Figure S3.** Pearson correlations among the urinary VOCs.

**Figure S4.** LASSO regression results. (A) Cross-validation plot showing binomial deviance versus log(λ). The red dots indicate the mean deviance, and the vertical dotted lines represent the λ values selected by cross-validation. (B) Lasso coefficient paths for different log(λ) values, illustrating variable shrinkage as regularization increases. LASSO, least absolute shrinkage and selection operator.

**Figure S5.** RCS plots of the association between the concentration of urinary VOCs and hyperlipidemia, stratified by sex: (A) Male, (B) Female.

**Figure S6.** RCS plots of the association between the concentration of urinary VOCs and hyperlipidemia, stratified by age group: (A) Age < 60 years, (B) Age ≥ 60 years.

**Figure S7.** WQS model results for the association between VOCs mixtures (ln-transformed) and hyperlipidemia, stratified by sex and age group. Panels A–D display results from the positive-direction WQS model, and panels E–H display results from the negative-direction WQS model. Panels (A) & (E): males; (B) & (F): females; (C) & (G): age < 60 years; (D) & (H): age ≥ 60 years. The model was adjusted for covariates including sex, age, race/ethnicity, PIR, education level, marital status, BMI, smoking status, alcohol consumption, and urinary creatinine level. WQS, weighted quantile sum; VOCs, volatile organic compounds.

**Figure S8.** Qgcomp model results for the association between VOCs mixtures and hyperlipidemia, stratified by sex and age group: (A) Male, (B) Female, (C) Age < 60 years, (D) Age ≥ 60 years.

**Figure S9** Trace plots of the beta1 parameters during MCMC sampling in the BKMR model.

**Figure S10.** Using the BKMR model, the overall relationship between VOCs mixtures and hyperlipidemia was stratified by sex and age group: (A) Male, (B) Female, (C) Age < 60 years, (D) Age ≥ 60 years.

**Figure S11.** The univariate dose-response relationship between each VOC and hyperlipidemia, evaluated using the BKMR model, with all other VOCs fixed at the 50th percentile, and stratified by sex and age group: (A) Male, (B) Female, (C) Age < 60 years, (D) Age ≥ 60 years.

**Figure S12.** The single-exposure risk of each VOC on hyperlipidemia, evaluated using the BKMR model, with all other VOCs fixed at the 25th, 50th, or 75th percentile, and stratified by sex and age group: (A) Male, (B) Female, (C) Age < 60 years, (D) Age ≥ 60 years.

**Figure S13.** The response of a single VOC, with the second VOC fixed at various percentiles (25th, 50th, 75th), and the remaining VOCs fixed at the 50th percentile, evaluated using the BKMR model and stratified by sex and age group: (A) Male, (B) Female, (C) Age < 60 years, (D) Age ≥ 60 years.

**Figure S14** Mediation effects of a single inflammatory marker in the association between urinary VOCs and hyperlipidemia.

Table S1 Distribution of urinary VOC metabolites (ng/mL) among US adults.

| Variable Name | Analyte Name | LOD (ng/mL) | Detection Rate (%) | Mean | Min | Percentile  25th | Percentile  50th | Percentile  75th | Max |
| --- | --- | --- | --- | --- | --- | --- | --- | --- | --- |
| MHA2 | 2-methylhippuric acid | 5.00 | 92.52% | 66.57 | 3.54 | 13.40 | 34.10 | 81.55 | 1660.00 |
| MHA3. MHA4 | 3- methylhippuric acid & 4- methylhippuric acid | 8.00 | 99.60% | 485.27 | 5.66 | 96.60 | 243.00 | 611.00 | 9920.00 |
| AAMA | N-acetyl-S-(2-carbamoylethyl)-L-cysteine | 2.20 | 99.95% | 94.03 | 1.56 | 27.90 | 53.30 | 106.00 | 2750.00 |
| AMCC | N-acetyl-S-(N-methylcarbamoyl)-L-cysteine | 6.26 | 99.90% | 263.45 | 4.43 | 82.75 | 162.00 | 320.00 | 3290.00 |
| ATCA | 2-aminothiazoline-4-carboxylic acid | 15.00 | 96.97% | 163.16 | 10.60 | 51.15 | 105.00 | 202.00 | 2090.00 |
| BMA | N-acetyl-S-(benzyl)-L-cysteine | 0.50 | 99.60% | 15.11 | 0.35 | 3.61 | 6.80 | 13.40 | 1040.00 |
| CEMA | N-acetyl-S-(2-carboxyethyl)-L-cysteine | 6.96 | 99.39% | 153.02 | 4.92 | 52.65 | 99.90 | 186.00 | 2240.00 |
| CYMA | N-acetyl-S-(2-cyanoethyl)-L-cysteine | 0.50 | 87.97% | 47.73 | 0.35 | 0.88 | 1.73 | 14.60 | 1620.00 |
| DHBMA | N-acetyl-S-(3, 4-dihidroxybutyl)-L-cysteine | 5.25 | 100.0% | 379.90 | 17.10 | 192.50 | 317.00 | 498.00 | 3890.00 |
| HPMA2 | N-acetyl-S-(2-hydroxypropyl)-L-cysteine | 5.30 | 96.72% | 70.42 | 3.75 | 17.15 | 32.30 | 61.75 | 9360.00 |
| HPMA3 | N-acetyl-S- (3-hydroxypropyl)-L-cysteine | 13.00 | 99.90% | 435.60 | 9.20 | 120.00 | 220.00 | 438.00 | 12100.00 |
| HPMMA | N-acetyl-S-(3-hydroxypropyl-1-methyl)-L-cysteine | 1.70 | 100.0% | 431.30 | 12.30 | 121.00 | 214.00 | 384.50 | 11100.00 |
| MHBMA3 | N-acetyl-S-(4-hydroxy-2-butenyl)-L-cysteine | 0.60 | 97.17% | 10.77 | 0.42 | 2.36 | 4.40 | 8.86 | 163.00 |
| PGA | Phenylglyoxylic acid | 12.00 | 99.85% | 281.80 | 8.50 | 132.00 | 221.00 | 360.50 | 2080.00 |
| MA | Mandelic acid | 12.00 | 99.04% | 190.88 | 8.50 | 78.75 | 133.00 | 229.50 | 5170.00 |

LOD: Limit of Detection.

Table S2 VOC metabolites and their parent compounds.

| Parent Compound | Analyte Name | Variable Name |
| --- | --- | --- |
| Acrolein | N-acetyl-S-(2-carboxyethyl)-L-cysteine | CEMA |
|  | N-acetyl-S- (3-hydroxypropyl)-L-cysteine | HPMA3 |
| Acrylamide | N-acetyl-S-(2-carbamoylethyl)-L-cysteine | AAMA |
| Acrylonitrile | N-acetyl-S-(2-cyanoethyl)-L-cysteine | CYMA |
| 1,3-Butadiene | N-acetyl-S-(3, 4-dihidroxybutyl)-L-cysteine | DHBMA |
|  | N-acetyl-S-(4-hydroxy-2-butenyl)-L-cysteine | MHBMA3 |
| Crotonaldehyde | N-acetyl-S-(3-hydroxypropyl-1-methyl)-L-cysteine | HPMMA |
| Cyanide | 2-aminothiazoline-4-carboxylic acid | ATCA |
| N, N-Dimethylformamide | N-acetyl-S-(N-methylcarbamoyl)-L-cysteine | AMCC |
| Ethylbenzene, styrene | Phenylglyoxylic acid | PGA |
| Propylene oxide | N-acetyl-S-(2-hydroxypropyl)-L-cysteine | HPMA2 |
| Styrene | Mandelic acid | MA |
| Toluene | N-acetyl-S-(benzyl)-L-cysteine | BMA |
| Xylene | 2-Methylhippuric acid | MHA2 |
|  | 3- methylhippuric acid & 4- methylhippuric acid | MHA3. MHA4 |

Table S3 Sex-stratified associations between individual urinary VOCs and hyperlipidemia among US adults.

| Variables | Q1 |  | Q2 | |  | Q3 | |  | Q4 | |  |  | Continuous | |  |
| --- | --- | --- | --- | --- | --- | --- | --- | --- | --- | --- | --- | --- | --- | --- | --- |
|  |  |  | OR (95%CI) | *p* value |  | OR (95%CI) | *p* value |  | OR (95%CI) | *p* value |  | *p* trend | OR (95%CI) | *p* value | adj_*p* value |
| MHA2 |  |  |  |  |  |  |  |  |  |  |  |  |  |  |  |
| Male | reference |  | 1.037 (0.670, 1.606) | 0.869 |  | 1.233 (0.712, 2.133) | 0.448 |  | 1.019 (0.627, 1.659) | 0.937 |  | 0.757 | 0.989 (0.835, 1.172) | 0.897 | 0.912 |
| Female | reference |  | 0.982 (0.573, 1.684) | 0.946 |  | 1.611 (0.944, 2.749) | 0.080 |  | 1.751 (1.156, 2.653) | **0.009** |  | **0.002** | 1.268 (1.113, 1.445) | **0.001** | **0.003** |
| MHA3. MHA4 |  |  |  |  |  |  |  |  |  |  |  |  |  |  |  |
| Male | reference |  | 1.467 (0.844, 2.550) | 0.171 |  | 1.390 (0.789, 2.450) | 0.249 |  | 1.021 (0.622, 1.677) | 0.933 |  | 0.890 | 1.018 (0.853, 1.214) | 0.843 | 0.912 |
| Female | reference |  | 1.297 (0.725, 2.320) | 0.374 |  | 1.254 (0.779, 2.017) | 0.345 |  | 1.811 (1.127, 2.912) | **0.015** |  | **0.018** | 1.251 (1.092, 1.434) | **0.002** | **0.005** |
| AAMA |  |  |  |  |  |  |  |  |  |  |  |  |  |  |  |
| Male | reference |  | 1.597 (0.989, 2.581) | 0.055 |  | 1.345 (0.849, 2.129) | 0.202 |  | 0.889 (0.546, 1.448) | 0.631 |  | 0.340 | 0.847 (0.676, 1.062) | 0.147 | 0.478 |
| Female | reference |  | 0.949 (0.578, 1.559) | 0.834 |  | 0.386 (0.229, 0.649) | **0.001** |  | 0.681 (0.430, 1.079) | 0.100 |  | **0.015** | 0.808 (0.649, 1.005) | 0.055 | 0.089 |
| AMCC |  |  |  |  |  |  |  |  |  |  |  |  |  |  |  |
| Male | reference |  | 1.499 (0.887, 2.533) | 0.128 |  | 1.744 (0.957, 3.176) | 0.069 |  | 1.769 (1.100, 2.846) | **0.020** |  | **0.031** | 1.229 (0.984, 1.536) | 0.069 | 0.478 |
| Female | reference |  | 1.567 (1.004, 2.446) | **0.048** |  | 1.146 (0.746, 1.761) | 0.527 |  | 2.397 (1.448, 3.969) | **0.001** |  | **0.007** | 1.501 (1.206, 1.869) | **<0.001** | **0.003** |
| ATCA |  |  |  |  |  |  |  |  |  |  |  |  |  |  |  |
| Male | reference |  | 1.156 (0.696, 1.921) | 0.569 |  | 0.938 (0.618, 1.424) | 0.761 |  | 0.970 (0.601, 1.566) | 0.901 |  | 0.695 | 1.010 (0.842, 1.212) | 0.913 | 0.912 |
| Female | reference |  | 0.829 (0.516, 1.331) | 0.430 |  | 0.809 (0.481, 1.364) | 0.420 |  | 1.516 (1.009, 2.280) | **0.046** |  | 0.136 | 1.163 (0.973, 1.391) | 0.096 | 0.124 |
| BMA |  |  |  |  |  |  |  |  |  |  |  |  |  |  |  |
| Male | reference |  | 1.277 (0.799, 2.040) | 0.301 |  | 1.125 (0.622, 2.032) | 0.693 |  | 1.049 (0.690, 1.596) | 0.818 |  | 0.946 | 1.023 (0.874, 1.198) | 0.744 | 0.912 |
| Female | reference |  | 0.798 (0.476, 1.338) | 0.386 |  | 1.029 (0.613, 1.726) | 0.913 |  | 0.766 (0.448, 1.309) | 0.323 |  | 0.508 | 0.945 (0.785, 1.138) | 0.546 | 0.591 |
| CEMA |  |  |  |  |  |  |  |  |  |  |  |  |  |  |  |
| Male | reference |  | 1.319 (0.730, 2.381) | 0.353 |  | 1.376 (0.870, 2.178) | 0.169 |  | 1.313 (0.741, 2.327) | 0.345 |  | 0.320 | 1.246 (0.929, 1.671) | 0.140 | 0.478 |
| Female | reference |  | 1.338 (0.807, 2.219) | 0.254 |  | 1.556 (1.020, 2.374) | **0.041** |  | 1.740 (1.168, 2.591) | **0.007** |  | **0.004** | 1.372 (1.144, 1.644) | **0.001** | **0.003** |
| CYMA |  |  |  |  |  |  |  |  |  |  |  |  |  |  |  |
| Male | reference |  | 0.750 (0.475, 1.183) | 0.211 |  | 0.853 (0.500, 1.455) | 0.554 |  | 0.889 (0.519, 1.521) | 0.662 |  | 0.755 | 0.993 (0.910, 1.083) | 0.870 | 0.912 |
| Female | reference |  | 1.581 (0.933, 2.678) | 0.087 |  | 1.025 (0.588, 1.786) | 0.929 |  | 1.844 (1.135, 2.995) | **0.014** |  | 0.083 | 1.126 (1.038, 1.222) | **0.005** | **0.010** |
| HPMA2 |  |  |  |  |  |  |  |  |  |  |  |  |  |  |  |
| Male | reference |  | 1.021 (0.573, 1.820) | 0.943 |  | 1.046 (0.589, 1.859) | 0.876 |  | 0.904 (0.545, 1.499) | 0.690 |  | 0.737 | 0.866 (0.720, 1.043) | 0.126 | 0.478 |
| Female | reference |  | 0.988 (0.604, 1.619) | 0.962 |  | 1.027 (0.667, 1.581) | 0.904 |  | 0.976 (0.583, 1.633) | 0.925 |  | 0.960 | 0.958 (0.775, 1.183) | 0.684 | 0.683 |
| HPMMA |  |  |  |  |  |  |  |  |  |  |  |  |  |  |  |
| Male | reference |  | 1.283 (0.723, 2.274) | 0.388 |  | 1.175 (0.759, 1.819) | 0.463 |  | 1.281 (0.743, 2.209) | 0.367 |  | 0.440 | 1.073 (0.844, 1.364) | 0.559 | 0.912 |
| Female | reference |  | 1.081 (0.689, 1.696) | 0.730 |  | 1.014 (0.628, 1.636) | 0.954 |  | 1.476 (1.003, 2.172) | **0.048** |  | 0.109 | 1.305 (1.100, 1.549) | **0.003** | **0.007** |
| MHBMA3 |  |  |  |  |  |  |  |  |  |  |  |  |  |  |  |
| Male | reference |  | 1.029 (0.616, 1.720) | 0.911 |  | 1.399 (0.823, 2.379) | 0.211 |  | 1.103 (0.622, 1.956) | 0.734 |  | 0.500 | 1.041 (0.845, 1.281) | 0.704 | 0.912 |
| Female | reference |  | 0.777 (0.442, 1.367) | 0.375 |  | 0.916 (0.584, 1.435) | 0.697 |  | 1.190 (0.756, 1.874) | 0.446 |  | 0.343 | 1.210 (1.054, 1.389) | **0.007** | **0.013** |
| PGA |  |  |  |  |  |  |  |  |  |  |  |  |  |  |  |
| Male | reference |  | 0.606 (0.354, 1.037) | 0.067 |  | 1.143 (0.680, 1.920) | 0.608 |  | 0.882 (0.530, 1.469) | 0.625 |  | 0.691 | 1.054 (0.868, 1.280) | 0.592 | 0.912 |
| Female | reference |  | 1.014 (0.573, 1.794) | 0.962 |  | 1.140 (0.686, 1.895) | 0.608 |  | 1.515 (0.889, 2.581) | 0.124 |  | 0.107 | 1.122 (0.898, 1.402) | 0.304 | 0.359 |
| MA |  |  |  |  |  |  |  |  |  |  |  |  |  |  |  |
| Male | reference |  | 1.193 (0.654, 2.175) | 0.560 |  | 0.911 (0.538, 1.541) | 0.723 |  | 1.280 (0.716, 2.291) | 0.398 |  | 0.621 | 1.144 (0.797, 1.642) | 0.460 | 0.912 |
| Female | reference |  | 0.814 (0.502, 1.321) | 0.399 |  | 0.786 (0.486, 1.270) | 0.319 |  | 1.229 (0.767, 1.967) | 0.385 |  | 0.449 | 1.268 (0.973, 1.654) | 0.078 | 0.112 |

N (male) = 1,023; N (female) = 956. The model was adjusted for covariates including sex, age, race/ethnicity, PIR, education level, marital status, BMI, smoking status, alcohol consumption, and urinary creatinine level.

Table S4 Age-stratified associations between individual urinary VOCs and hyperlipidemia among US adults.

| Variables | Q1 |  | Q2 | |  | Q3 | |  | Q4 | |  |  | Continuous | |  |
| --- | --- | --- | --- | --- | --- | --- | --- | --- | --- | --- | --- | --- | --- | --- | --- |
|  |  |  | OR (95%CI) | *p* value |  | OR (95%CI) | *p* value |  | OR (95%CI) | *p* value |  | *p* trend | OR (95%CI) | *p* value | adj_*p* value |
| MHA2 |  |  |  |  |  |  |  |  |  |  |  |  |  |  |  |
| Age<60 | reference |  | 0.972 (0.648, 1.460) | 0.891 |  | 1.138 (0.706, 1.837) | 0.590 |  | 1.404 (0.917, 2.150) | 0.117 |  | 0.098 | 1.132 (0.995, 1.289) | 0.059 | 0.109 |
| Age≥60 | reference |  | 1.037 (0.617, 1.741) | 0.890 |  | 1.997 (0.607, 3.603) | 0.323 |  | 1.228 (0.741, 2.036) | 0.419 |  | 0.155 | 1.125 (0.949, 1.333) | 0.172 | 0.570 |
| MHA3. MHA4 |  |  |  |  |  |  |  |  |  |  |  |  |  |  |  |
| Age<60 | reference |  | 1.541 (0.946, 2.512) | 0.081 |  | 1.171 (0.718, 1.910) | 0.520 |  | 1.585 (1.033, 2.430) | **0.035** |  | 0.104 | 1.153 (1.013, 1.313) | **0.032** | **0.039** |
| Age≥60 | reference |  | 1.065 (0.612, 1.852) | 0.820 |  | 1.452 (0.795, 2.651) | 0.220 |  | 1.439 (0.816, 2.539) | 0.204 |  | 0.097 | 1.099 (0.914, 1.321) | 0.310 | 0.587 |
| AAMA |  |  |  |  |  |  |  |  |  |  |  |  |  |  |  |
| Age<60 | reference |  | 1.044 (0.717, 1.520) | 0.820 |  | 0.696 (0.468, 1.033) | 0.072 |  | 0.717 (0.475, 1.081) | 0.110 |  | **0.029** | 0.839 (0.713, 0.989) | **0.037** | **0.039** |
| Age≥60 | reference |  | 2.106 (0.523, 3.947) | 0.621 |  | 1.068 (0.555, 2.056) | 0.840 |  | 1.038 (0.535, 2.014) | 0.911 |  | 0.449 | 0.766 (0.538, 1.091) | 0.137 | 0.570 |
| AMCC |  |  |  |  |  |  |  |  |  |  |  |  |  |  |  |
| Age<60 | reference |  | 1.935 (1.307, 2.866) | **0.001** |  | 1.551 (0.941, 2.554) | 0.084 |  | 2.502 (1.628, 3.844) | **<0.001** |  | **0.001** | 1.434 (1.194, 1.722) | **<0.001** | **0.001** |
| Age≥60 | reference |  | 1.194 (0.641, 2.222) | 0.570 |  | 1.078 (0.607, 1.915) | 0.794 |  | 1.608 (0.784, 3.299) | 0.191 |  | 0.216 | 1.184 (0.845, 1.658) | 0.321 | 0.587 |
| ATCA |  |  |  |  |  |  |  |  |  |  |  |  |  |  |  |
| Age<60 | reference |  | 1.253 (0.794, 1.976) | 0.326 |  | 0.881 (0.545, 1.426) | 0.601 |  | 1.470 (0.954, 2.266) | 0.079 |  | 0.265 | 1.117 (0.968, 1.289) | 0.127 | 0.183 |
| Age≥60 | reference |  | 0.609 (0.319, 1.163) | 0.130 |  | 0.740 (0.401, 1.367) | 0.330 |  | 1.471 (0.792, 2.732) | 0.217 |  | 0.142 | 1.143 (0.926, 1.411) | 0.208 | 0.570 |
| BMA |  |  |  |  |  |  |  |  |  |  |  |  |  |  |  |
| Age<60 | reference |  | 1.279 (0.846, 1.934) | 0.238 |  | 1.367 (0.855, 2.185) | 0.188 |  | 1.286 (0.897, 1.845) | 0.168 |  | 0.161 | 1.001 (0.856, 1.171) | 0.989 | 0.989 |
| Age≥60 | reference |  | 0.588 (0.285, 1.212) | 0.147 |  | 0.829 (0.364, 1.891) | 0.651 |  | 0.847 (0.361, 1.986) | 0.697 |  | 0.973 | 1.035 (0.791, 1.355) | 0.797 | 0.905 |
| CEMA |  |  |  |  |  |  |  |  |  |  |  |  |  |  |  |
| Age<60 | reference |  | 1.437 (0.853, 2.420) | 0.169 |  | 1.869 (1.254, 2.785) | **0.003** |  | 1.700 (1.148, 2.518) | **0.009** |  | **0.003** | 1.480 (1.248, 1.754) | **<0.001** | **0.001** |
| Age≥60 | reference |  | 1.114 (0.545, 2.277) | 0.763 |  | 1.007 (0.585, 1.733) | 0.981 |  | 0.916 (0.508, 1.652) | 0.766 |  | 0.723 | 0.924 (0.682, 1.252) | 0.603 | 0.871 |
| CYMA |  |  |  |  |  |  |  |  |  |  |  |  |  |  |  |
| Age<60 | reference |  | 0.763 (0.519, 1.120) | 0.164 |  | 0.828 (0.531, 1.292) | 0.399 |  | 1.197 (0.780, 1.837) | 0.403 |  | 0.382 | 1.051 (0.985, 1.122) | 0.134 | 0.183 |
| Age≥60 | reference |  | 1.727 (0.957, 3.118) | 0.069 |  | 0.968 (0.517, 1.812) | 0.917 |  | 1.923 (0.112, 3.323) | 0.420 |  | 0.165 | 1.008 (0.885, 1.147) | 0.906 | 0.905 |
| HPMA2 |  |  |  |  |  |  |  |  |  |  |  |  |  |  |  |
| Age<60 | reference |  | 0.913 (0.605, 1.379) | 0.661 |  | 0.936 (0.581, 1.507) | 0.781 |  | 1.092 (0.719, 1.657) | 0.675 |  | 0.679 | 1.015 (0.863, 1.195) | 0.851 | 0.851 |
| Age≥60 | reference |  | 1.275 (0.738, 2.204) | 0.377 |  | 1.391 (0.822, 2.354) | 0.213 |  | 0.619 (0.322, 1.191) | 0.147 |  | 0.171 | 0.730 (0.554, 1.961) | 0.326 | 0.333 |
| HPMMA |  |  |  |  |  |  |  |  |  |  |  |  |  |  |  |
| Age<60 | reference |  | 1.118 (0.684, 1.829) | 0.651 |  | 1.020 (0.692, 1.503) | 0.919 |  | 1.426 (0.929, 2.190) | 0.103 |  | 0.187 | 1.220 (1.036, 1.436) | **0.018** | **0.048** |
| Age≥60 | reference |  | 2.701 (0.293, 5.646) | 0.259 |  | 1.455 (0.668, 3.168) | 0.338 |  | 1.955 (0.976, 3.913) | 0.058 |  | 0.223 | 1.022 (0.743, 1.406) | 0.893 | 0.905 |
| MHBMA3 |  |  |  |  |  |  |  |  |  |  |  |  |  |  |  |
| Age<60 | reference |  | 0.911 (0.590, 1.406) | 0.668 |  | 1.017 (0.684, 1.514) | 0.931 |  | 1.199 (0.778, 1.847) | 0.405 |  | 0.358 | 1.156 (1.016, 1.315) | **0.029** | **0.049** |
| Age≥60 | reference |  | 0.807 (0.387, 1.683) | 0.561 |  | 1.593 (0.780, 3.255) | 0.197 |  | 0.913 (0.433, 1.927) | 0.808 |  | 0.644 | 0.949 (0.723, 1.244) | 0.698 | 0.905 |
| PGA |  |  |  |  |  |  |  |  |  |  |  |  |  |  |  |
| Age<60 | reference |  | 0.850 (0.529, 1.367) | 0.497 |  | 0.922 (0.593, 1.433) | 0.713 |  | 1.116 (0.673, 1.850) | 0.666 |  | 0.584 | 1.017 (0.843, 1.228) | 0.855 | 0.926 |
| Age≥60 | reference |  | 0.920 (0.515, 1.646) | 0.776 |  | 1.101 (0.585, 2.074) | 0.761 |  | 1.423 (0.748, 2.706) | 0.277 |  | 0.230 | 1.227 (0.882, 1.707) | 0.219 | 0.570 |
| MA |  |  |  |  |  |  |  |  |  |  |  |  |  |  |  |
| Age<60 | reference |  | 0.906 (0.572, 1.437) | 0.671 |  | 0.864 (0.558, 1.337) | 0.506 |  | 1.269 (0.834, 1.933) | 0.261 |  | 0.352 | 1.210 (0.937, 1.563) | 0.141 | 0.183 |
| Age≥60 | reference |  | 1.042 (0.548, 1.981) | 0.897 |  | 0.934 (0.377, 2.313) | 0.880 |  | 1.394 (0.623, 3.118) | 0.412 |  | 0.487 | 1.249 (0.770, 2.026) | 0.361 | 0.587 |

N (Age<60) = 1,327; N (Age≥60) = 652. The model was adjusted for covariates including sex, age, race/ethnicity, PIR, education level, marital status, BMI, smoking status, alcohol consumption, and urinary creatinine level.

Table S5 Associations between the WQS regression index and hyperlipidemia among US adults.

| Assumption | WQS | Estimate | Standard Error | Z-value | *P-*value | OR (95%CI) |
| --- | --- | --- | --- | --- | --- | --- |
| **Positive** | Overall | 0.271 | 0.102 | 2.640 | **0.008** | 1.312 (1.073, 1.606) |
|  | Male | 0.115 | 0.137 | 0.841 | 0.400 | 1.122 (0.857, 1.471) |
|  | Female | 0.414 | 0.172 | 2.394 | **0.017** | 1.510 (1.078, 2.118) |
|  | Age<60 | 0.309 | 0.118 | 2.614 | **0.009** | 1.363 (1.080, 1.717) |
|  | Age≥60 | 0.102 | 0.181 | 0.563 | 0.573 | 1.108 (0.776, 1.581) |
| **Negative** | Overall | -0.203 | 0.096 | -2.219 | **0.033** | 0.816 (0.677, 0.984) |
|  | Male | -0.051 | 0.126 | -0.402 | 0.688 | 0.950 (0.742, 1.218) |
|  | Female | -0.360 | 0.164 | -2.197 | **0.028** | 0.698 (0.505, 0.963) |
|  | Age<60 | -0.323 | 0.315 | -2.396 | **0.016** | 0.723 (0.555, 0.943) |
|  | Age≥60 | -0.101 | 0.175 | -0.578 | 0.563 | 0.904 (0.641, 1.274) |

N = 1979, The model was adjusted for covariates including sex, age, race/ethnicity, PIR, education level, marital

status, BMI, smoking status, alcohol consumption, and urinary creatinine level.

Table S6 Associations between urinary VOCs and hyperlipidemia among US adults: A quantile g-computation approach.

| Qgcomp | Estimate | Standard Error | Z-value | *P-*value | OR (95%CI) |
| --- | --- | --- | --- | --- | --- |
| Overall | 0.260 | 0.115 | 2.263 | **0.024** | 1.296 (1.035,1.623) |
| Male | -0.020 | 0.124 | -0.161 | 0.872 | 0.980 (0.769,1.249) |
| Female | 0.378 | 0.134 | 2.831 | **0.005** | 1.460 (1.123,1.897) |
| Age<60 | 0.259 | 0.115 | 2.263 | **0.024** | 1.296 (1.035,1.623) |
| Age≥60 | -0.002 | 0.162 | -0.014 | 0.989 | 0.998 (0.726,1.371) |

N = 1979, The model was adjusted for covariates including sex, age, race/ethnicity, PIR, education

level, marital status, BMI, smoking status, alcohol consumption, and urinary creatinine level.

Table S7 Inflammation markers as multiple mediators in the association between VOCs mixture and hyperlipidemia among US adults.

| Multi-mediator model | ACME | ADE | Total Effect | Prop. Mediated | *P*-value |
| --- | --- | --- | --- | --- | --- |
| VOCs mixture →Hyperlipidemia(ACME) | 0.032(0.013,0.051) | —— | —— | —— | **0.001** |
| VOCs mixture →Hyperlipidemia(ADE) | —— | 0.127(0.027,0.227) | —— | —— | **0.013** |
| VOCs mixture →Hyperlipidemia(Total Effect) | —— | —— | 0.159(0.059,0.259) | —— | **0.002** |
| VOCs mixture →WBC count →Hyperlipidemia | 0.024(0.009,0.039) | —— | —— | 15.094% | **0.002** |
| VOCs mixture →Lymphocyte count →Hyperlipidemia | 0.007(-0.006,0.019) | —— | —— | 4.403% | 0.305 |
| VOCs mixture →Neutrophil count →Hyperlipidemia | 0.001(-0.003,0.006) | —— | —— | 0.630% | 0.565 |

N = 1979, The model was adjusted for covariates including sex, age, race/ethnicity, PIR, education level, marital status, BMI, smoking status, alcohol consumption, and urinary creatinine level.

Table S8 Inflammation markers as single mediators in the association between VOCs mixture and hyperlipidemia among US adults.

| Single mediator model | ACME | ADE | Total Effect | Prop. Mediated | *P*-value |
| --- | --- | --- | --- | --- | --- |
| VOCs mixture →WBC count →Hyperlipidemia | 0.008(0.003,0.010) | 0.046(0.012,0.080) | 0.054(0.021,0.090) | 15.285% | **<0.001** |
| VOCs mixture →Lymphocyte count →Hyperlipidemia | 0.007(0.003,0.010) | 0.046(0.014,0.080) | 0.054(0.022,0.090) | 14.528% | **0.002** (1.035,1.623) |
| VOCs mixture →Neutrophil count →Hyperlipidemia | 0.004(0.000,0.010) | 0.050(0.014,0.080) | 0.054(0.019,0.090) | 7.510% | **0.044** (0.726,1.371) |

N = 1979, The model was adjusted for covariates including sex, age, race/ethnicity, PIR, education level, marital status, BMI, smoking status, alcohol consumption, and urinary creatinine level.

Table S9 Sensitivity analysis of the association between VOCs and hyperlipidemia without urinary creatinine adjustment.

| Variables | Q1 |  | Q2 | |  | Q3 | |  | Q4 | |  |  | | Continuous | | |  |
| --- | --- | --- | --- | --- | --- | --- | --- | --- | --- | --- | --- | --- | --- | --- | --- | --- | --- |
|  |  |  | OR (95%CI) | *p* value |  | OR (95%CI) | *p* value |  | OR (95%CI) | *p* value | |  | *p* trend | | OR (95%CI) | *p* value | *adj_p value* |
| MHA2 |  |  |  |  |  |  |  |  |  |  | |  |  | |  |  |  |
| Overall | reference |  | 1.547(1.073, 2.230) | **0.020** |  | 1.168 (0.822, 1.660) | 0.380 |  | 1.591 (1.111, 2.279) | **0.012** | |  | **0.037** | | 1.131 (1.028,1.243) | **0.012** | **0.043** |
| MHA3. MHA4 |  |  |  |  |  |  |  |  |  |  | |  |  | |  |  |  |
| Overall | reference |  | 1.448(0.973, 2.155) | 0.067 |  | 1.370 (0.947, 1.982) | 0.094 |  | 1.472(1.033, 2.097) | **0.033** | |  | **0.040** | | 1.125 (1.019,1.242) | **0.021** | **0.044** |
| AAMA |  |  |  |  |  |  |  |  |  |  | |  |  | |  |  |  |
| Overall | reference |  | 0.893(0.611, 1.304) | 0.551 |  | 0.881 (0.634, 1.224) | 0.445 |  | 0.778(0.572, 1.059) | 0.109 | |  | 0.098 | | 0.913 (0.824,1.012) | 0.082 | 0.129 |
| AMCC |  |  |  |  |  |  |  |  |  |  | |  |  | |  |  |  |
| Overall | reference |  | 1.427(1.020, 1.997) | **0.038** |  | 1.513 (0.998, 2.292) | 0.051 |  | 2.023(1.373, 2.981) | **0.001** | |  | **0.001** | | 1.256 (1.102,1.431) | **0.001** | **0.012** |
| ATCA |  |  |  |  |  |  |  |  |  |  | |  |  | |  |  |  |
| Overall | reference |  | 1.016(0.693, 1.488) | 0.935 |  | 1.261 (0.823, 1.931) | 0.281 |  | 1.236(0.798, 1.913) | 0.336 | |  | 0.226 | | 1.130 (0.979,1.304) | 0.094 | 0.129 |
| BMA |  |  |  |  |  |  |  |  |  |  | |  |  | |  |  |  |
| Overall | reference |  | 0.941(0.668, 1.327) | 0.725 |  | 1.168 (0.799, 1.710) | 0.416 |  | 1.115(0.785, 1.584) | 0.538 | |  | 0.345 | | 1.026 (0.903,1.167) | 0.686 | 0.686 |
| CEMA |  |  |  |  |  |  |  |  |  |  | |  |  | |  |  |  |
| Overall | reference |  | 1.123(0.793, 1.591) | 0.507 |  | 1.191 (0.864, 1.640) | 0.280 |  | 1.521(1.099, 2.105) | **0.012** | |  | **0.018** | | 1.196 (1.071,1.335) | **0.002** | **0.012** |
| CYMA |  |  |  |  |  |  |  |  |  |  | |  |  | |  |  |  |
| Overall | reference |  | 1.187(0.841, 1.675) | 0.323 |  | 0.955 (0.662, 1.379) | 0.804 |  | 1.278(0.940, 1.737) | 0.115 | |  | 0.325 | | 1.046 (0.991,1.103) | 0.099 | 0.129 |
| HPMA2 |  |  |  |  |  |  |  |  |  |  | |  |  | |  |  |  |
| Overall | reference |  | 0.824(0.581, 1.167) | 0.269 |  | 0.905 (0.676, 1.212) | 0.497 |  | 0.858(0.618, 1.190) | 0.353 | |  | 0.502 | | 0.955 (0.849,1.073) | 0.430 | 0.484 |
| HPMMA |  |  |  |  |  |  |  |  |  |  | |  |  | |  |  |  |
| Overall | reference |  | 1.290(0.856, 1.943) | 0.219 |  | 1.291 (0.924, 1.803) | 0.131 |  | 1.422(1.011, 2.000) | **0.043** | |  | **0.040** | | 1.144 (1.024,1.278) | **0.018** | 0.054 |
| MHBMA3 |  |  |  |  |  |  |  |  |  |  | |  |  | |  |  |  |
| Overall | reference |  | 0.830(0.581, 1.186) | 0.300 |  | 1.010 (0.746, 1.366) | 0.949 |  | 1.177(0.860, 1.610) | 0.302 | |  | 0.165 | | 1.104 (1.007,1.211) | **0.036** | 0.078 |
| PGA |  |  |  |  |  |  |  |  |  |  | |  |  | |  |  |  |
| Overall | reference |  | 0.828(0.561, 1.222) | 0.335 |  | 0.915 (0.638, 1.312) | 0.624 |  | 1.158(0.753, 1.780) | 0.498 | |  | 0.418 | | 1.065 (0.903,1.256) | 0.447 | 0.484 |
| MA |  |  |  |  |  |  |  |  |  |  | |  |  | |  |  |  |
| Overall | reference |  | 1.218(0.853, 1.738) | 0.272 |  | 1.229 (0.849, 1.779) | 0.268 |  | 1.302(0.909, 1.865) | 0.148 | |  | 0.164 | | 1.140 (0.975,1.333) | 0.098 | 0.129 |

N = 1979, The model was adjusted for covariates including sex, age, race/ethnicity, PIR, education level, marital status, BMI, smoking status, alcohol consumption, and urinary creatinine level.

Table S10 Sensitivity analysis of the relationship between urinary VOCs and hyperlipidemia after excluding individuals with urinary creatinine >300 mg/L or urinary creatinine <30 mg/L.

| Variables | Q1 |  | Q2 | |  | Q3 | |  | Q4 | |  |  | Continuous | | |  |
| --- | --- | --- | --- | --- | --- | --- | --- | --- | --- | --- | --- | --- | --- | --- | --- | --- |
|  |  |  | OR (95%CI) | *p* value |  | OR (95%CI) | *p* value |  | OR (95%CI) | *p* value |  | *p* trend | OR (95%CI) | *p* value | *adj_p value* | |
| MHA2 |  |  |  |  |  |  |  |  |  |  |  |  |  |  |  | |
| Overall | reference |  | 1.027 (0.756, 1.395) | 0.863 |  | 1.282 (0.860, 1.911) | 0.218 |  | 1.369 (0.983, 1.908) | 0.063 |  | **0.031** | 1.128 (1.014, 1.254) | **0.027** | **0.045** | |
| MHA3. MHA4 |  |  |  |  |  |  |  |  |  |  |  |  |  |  |  | |
| Overall | reference |  | 1.390 (0.943, 2.048) | 0.095 |  | 1.359 (0.891, 2.073) | 0.151 |  | 1.451 (1.026, 2.053) | **0.036** |  | **0.045** | 1.128 (1.012, 1.256) | **0.030** | **0.045** | |
| AAMA |  |  |  |  |  |  |  |  |  |  |  |  |  |  |  | |
| Overall | reference |  | 1.264 (0.857, 1.863) | 0.233 |  | 0.752 (0.537, 1.053) | 0.096 |  | 0.792 (0.561, 1.120) | 0.183 |  | 0.062 | 0.823 (0.707, 0.958) | **0.013** | 0.055 | |
| AMCC |  |  |  |  |  |  |  |  |  |  |  |  |  |  |  | |
| Overall | reference |  | 1.524 (1.024, 2.270) | **0.038** |  | 1.367 (0.953, 1.960) | 0.088 |  | 2.103 (1.423, 3.109) | **<0.001** |  | **<0.001** | 1.374 (1.157, 1.632) | **<0.001** | **0.006** | |
| ATCA |  |  |  |  |  |  |  |  |  |  |  |  |  |  |  | |
| Overall | reference |  | 1.162 (0.761, 1.776) | 0.480 |  | 0.899 (0.622, 1.300) | 0.567 |  | 1.727 (1.156, 2.580) | **0.008** |  | **0.031** | 1.166 (1.020, 1.333) | **0.025** | 0.065 | |
| BMA |  |  |  |  |  |  |  |  |  |  |  |  |  |  |  | |
| Overall | reference |  | 1.006 (0.708, 1.429) | 0.972 |  | 1.051 (0.695, 1.590) | 0.809 |  | 1.045 (0.746, 1.463) | 0.797 |  | 0.742 | 1.000 (0.872, 1.146) | 0.996 | 0.996 | |
| CEMA |  |  |  |  |  |  |  |  |  |  |  |  |  |  |  | |
| Overall | reference |  | 1.368 (0.893, 2.096) | 0.147 |  | 1.553 (1.186, 2.036) | **0.002** |  | 1.539 (1.048, 2.260) | **0.029** |  | **0.014** | 1.329 (1.125, 1.569) | **0.001** | **0.007** | |
| CYMA |  |  |  |  |  |  |  |  |  |  |  |  |  |  |  | |
| Overall | reference |  | 0.936 (0.688, 1.275) | 0.672 |  | 0.898 (0.654, 1.232) | 0.498 |  | 1.153 (0.841, 1.580) | 0.372 |  | 0.470 | 1.042 (0.983, 1.104) | 0.161 | 0.210 | |
| HPMA2 |  |  |  |  |  |  |  |  |  |  |  |  |  |  |  | |
| Overall | reference |  | 1.026 (0.731, 1.441) | 0.879 |  | 1.025 (0.692, 1.518) | 0.900 |  | 0.985 (0.657, 1.478) | 0.942 |  | 0.945 | 0.930 (0.801, 1.080) | 0.337 | 0.398 | |
| HPMMA |  |  |  |  |  |  |  |  |  |  |  |  |  |  |  | |
| Overall | reference |  | 1.112 (0.720, 1.719) | 0.626 |  | 1.064 (0.791, 1.430) | 0.678 |  | 1.399 (0.960, 2.039) | 0.079 |  | 0.100 | 1.176 (0.999, 1.385) | 0.052 | 0.096 | |
| MHBMA3 |  |  |  |  |  |  |  |  |  |  |  |  |  |  |  | |
| Overall | reference |  | 0.929 (0.632, 1.364) | 0.701 |  | 1.063 (0.763, 1.480) | 0.713 |  | 1.110 (0.774, 1.592) | 0.564 |  | 0.416 | 1.107 (0.972, 1.261) | 0.122 | 0.176 | |
| PGA |  |  |  |  |  |  |  |  |  |  |  |  |  |  |  | |
| Overall | reference |  | 0.746 (0.518, 1.075) | 0.113 |  | 0.983 (0.682, 1.417) | 0.925 |  | 1.088 (0.728, 1.626) | 0.675 |  | 0.385 | 1.048 (0.883, 1.243) | 0.589 | 0.638 | |
| MA |  |  |  |  |  |  |  |  |  |  |  |  |  |  |  | |
| Overall | reference |  | 0.932 (0.609, 1.427) | 0.742 |  | 1.063 (0.732, 1.543) | 0.744 |  | 1.203 (0.809, 1.788) | 0.355 |  | 0.279 | 1.207 (0.950, 1.535) | 0.121 | 0.176 | |

N = 1979, The model was adjusted for covariates including sex, age, race/ethnicity, PIR, education level, marital status, BMI, smoking status, alcohol consumption, and urinary creatinine level.

Table S11 Sensitivity analysis of the association between VOC and hyperlipidemia based on different smoking statuses.

| Variables |  | Current smokers | | |  | Former smokers | | |  | Never smokers | | |  |
| --- | --- | --- | --- | --- | --- | --- | --- | --- | --- | --- | --- | --- | --- |
|  |  | OR (95%CI) | *p* value | *adj_p value* |  | OR (95%CI) | *p* value | *adj_p value* |  | OR (95%CI) | *p* value | *adj_p value* |  |
| MHA2 |  |  |  |  |  |  |  |  |  |  |  |  |  |
| Overall |  | 1.178 (1.032, 1.246) | **0.017** | **0.003** |  | 1.165 (0.942, 1.441) | 0.156 | 0.508 |  | 1.279 (0.886, 1.846) | 0.185 | 0.482 |  |
| MHA3. MHA4 |  |  |  |  |  |  |  |  |  |  |  |  |  |
| Overall |  | 1.130 (1.049, 1.201) | **0.006** | **0.027** |  | 1.111 (0.882, 1.400) | 0.366 | 0.793 |  | 1.308 (0.888, 1.926) | 0.170 | 0.482 |  |
| AAMA |  |  |  |  |  |  |  |  |  |  |  |  |  |
| Overall |  | 0.651 (0.494, 0.859) | **0.003** | **0.013** |  | 0.946 (0.690, 1.299) | 0.729 | 0.863 |  | 0.663 (0.435, 1.012) | 0.056 | 0.375 |  |
| AMCC |  |  |  |  |  |  |  |  |  |  |  |  |  |
| Overall |  | 1.533 (1.199, 1.959) | **0.001** | **0.012** |  | 1.355 (0.905, 2.028) | 0.138 | 0.508 |  | 1.483 (0.987, 2.229) | 0.058 | 0.375 |  |
| ATCA |  |  |  |  |  |  |  |  |  |  |  |  |  |
| Overall |  | 1.126 (0.942, 1.345) | 0.188 | 0.271 |  | 1.079 (0.821, 1.418) | 0.577 | 0.842 |  | 1.097 (0.829, 1.451) | 0.511 | 0.558 |  |
| BMA |  |  |  |  |  |  |  |  |  |  |  |  |  |
| Overall |  | 1.074 (0.877, 1.315) | 0.482 | 0.570 |  | 0.963 (0.755, 1.227) | 0.754 | 0.863 |  | 0.914 (0.695, 1.203) | 0.515 | 0.558 |  |
| CEMA |  |  |  |  |  |  |  |  |  |  |  |  |  |
| Overall |  | 1.422 (1.135, 1.783) | **0.003** | **0.013** |  | 1.110 (0.761, 1.619) | 0.583 | 0.842 |  | 1.339 (0.911, 1.969) | 0.135 | 0.482 |  |
| CYMA |  |  |  |  |  |  |  |  |  |  |  |  |  |
| Overall |  | 1.000 (0.854, 1.171) | 0.999 | 0.999 |  | 1.119 (0.957, 1.309) | 0.155 | 0.508 |  | 0.994 (0.821, 1.204) | 0.949 | 0.949 |  |
| HPMA2 |  |  |  |  |  |  |  |  |  |  |  |  |  |
| Overall |  | 0.855 (0.701, 1.043) | 0.119 | 0.271 |  | 0.787 (0.615, 1.007) | 0.057 | 0.508 |  | 1.161 (0.767, 1.757) | 0.473 | 0.558 |  |
| HPMMA |  |  |  |  |  |  |  |  |  |  |  |  |  |
| Overall |  | 1.370 (0.900, 2.086) | 0.139 | 0.271 |  | 1.278 (0.827, 1.975) | 0.264 | 0.687 |  | 1.144 (0.870, 1.505) | 0.327 | 0.558 |  |
| MHBMA3 |  |  |  |  |  |  |  |  |  |  |  |  |  |
| Overall |  | 1.246 (0.917, 1.695) | 0.157 | 0.271 |  | 0.900 (0.617, 1.312) | 0.576 | 0.842 |  | 1.175 (0.891, 1.550) | 0.248 | 0.538 |  |
| PGA |  |  |  |  |  |  |  |  |  |  |  |  |  |
| Overall |  | 1.004 (0.778, 1.294) | 0.978 | 0.999 |  | 1.043 (0.753, 1.444) | 0.797 | 0.863 |  | 1.165 (0.777, 1.747) | 0.453 | 0.558 |  |
| MA |  |  |  |  |  |  |  |  |  |  |  |  |  |
| Overall |  | 1.319 (0.911, 1.909) | 0.140 | 0.271 |  | 0.982 (0.632, 1.526) | 0.935 | 0.935 |  | 1.189 (0.789, 1.793) | 0.401 | 0.558 |  |

N = 1979, The model was adjusted for covariates including sex, age, race/ethnicity, PIR, education level, marital status, BMI, smoking status,

alcohol consumption, and urinary creatinine level.


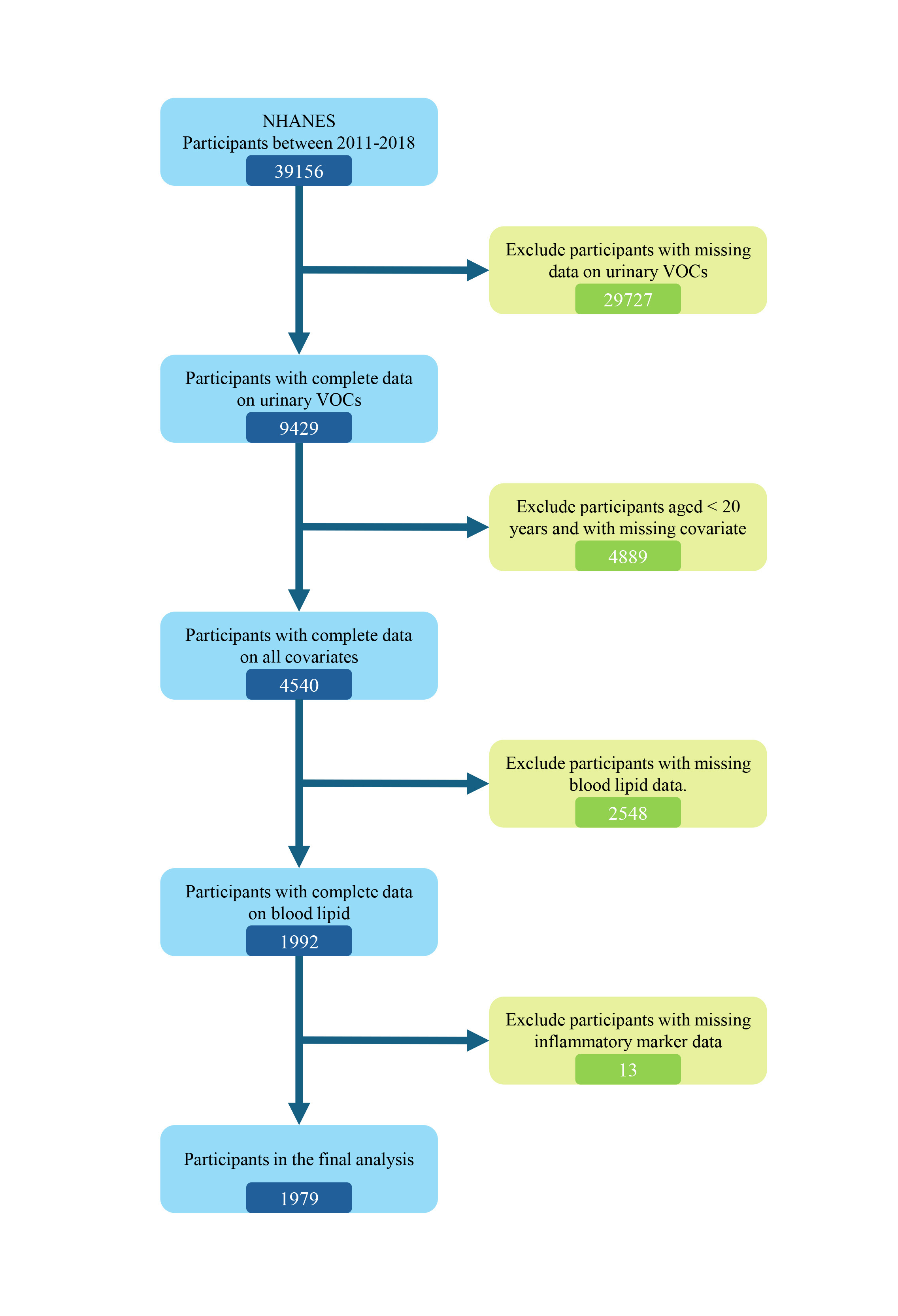


Figure S1. Flowchart for the selection of participants. NHANES, the National Health and Examination Survey; VOCs, volatile organic compound.


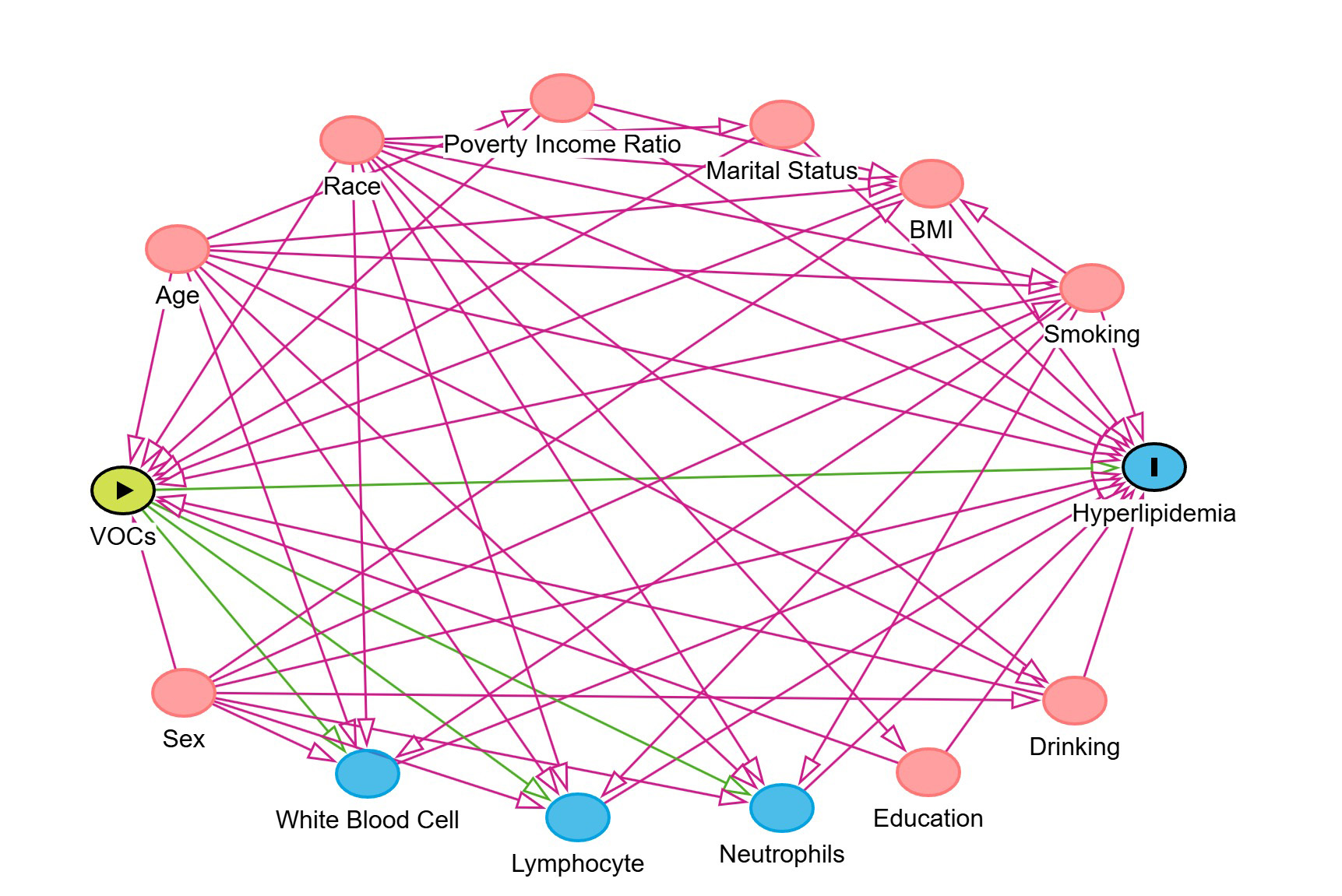


Figure S2 Covariate selection for evaluating the impact of urinary VOCs on hyperlipidemia was guided by a DAG. DAG, Directed Acyclic Graph; VOCs, volatile organic compounds.


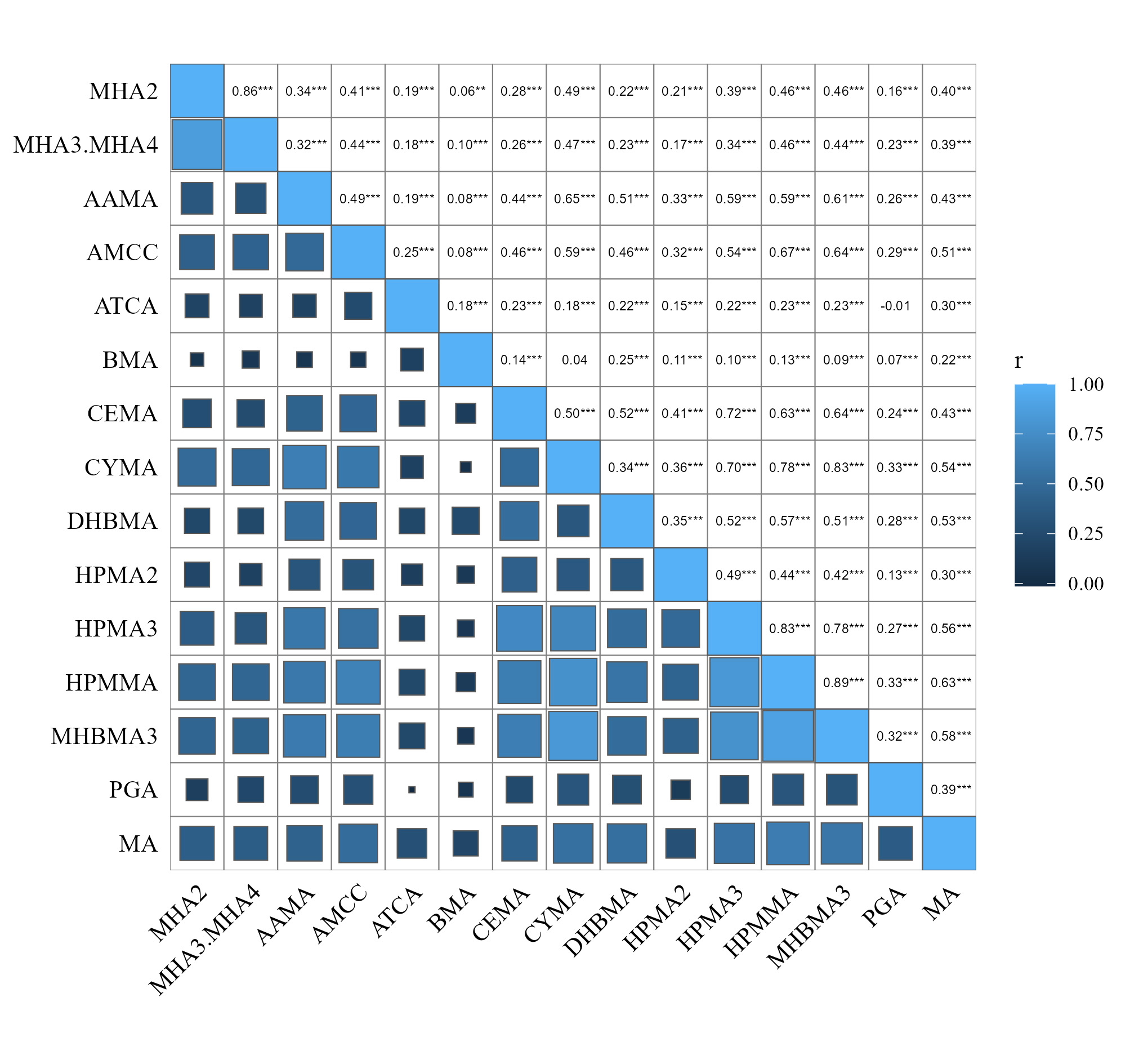


Figure S3 Pearson correlations among the urinary VOCs. VOCs, volatile organic compounds.


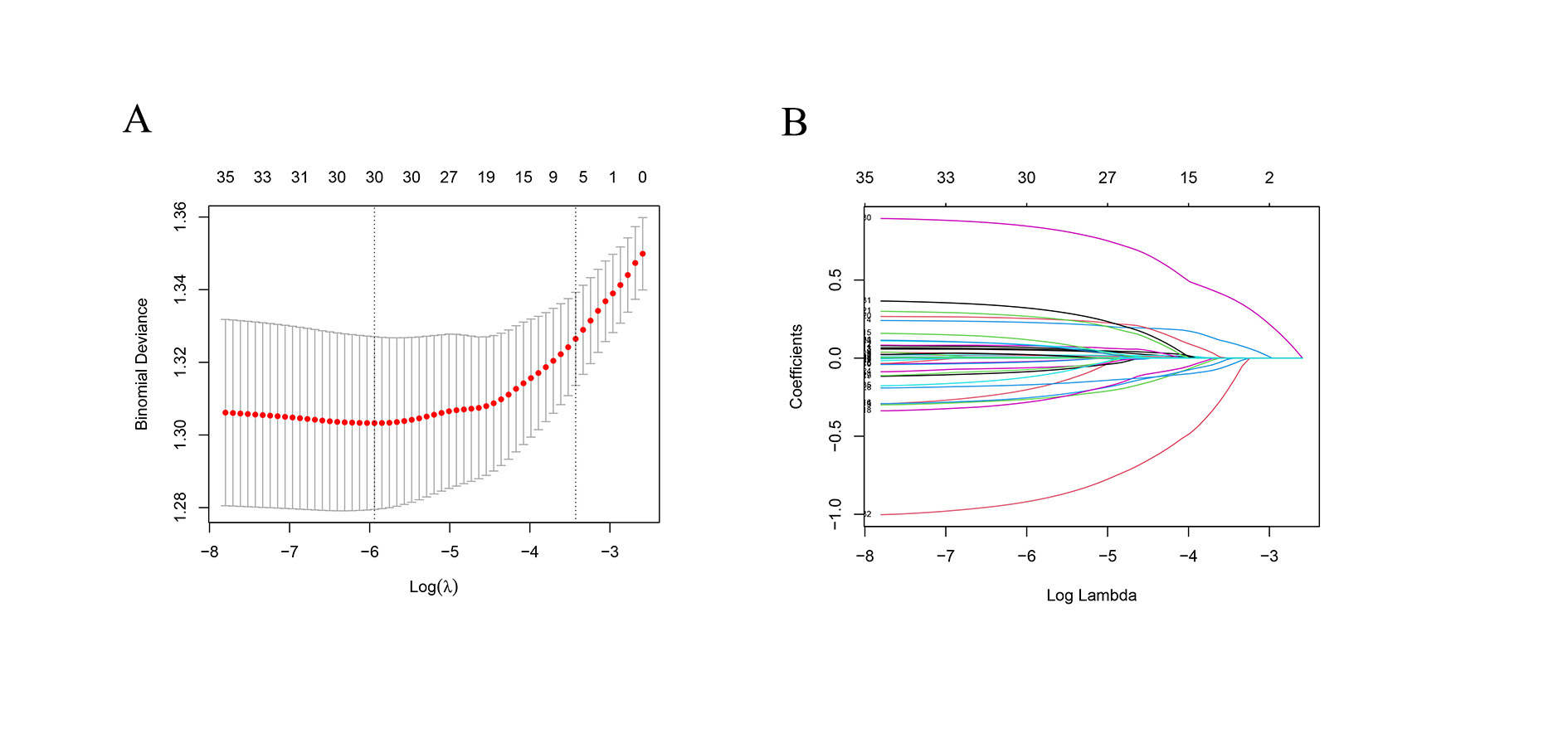


Figure S4 LASSO regression results. (A) Cross-validation plot showing binomial deviance versus log(λ). The red dots indicate the mean deviance, and the vertical dotted lines represent the λ values selected by cross-validation. (B) Lasso coefficient paths for different log(λ) values, illustrating variable shrinkage as regularization increases. LASSO, least absolute shrinkage and selection operator.


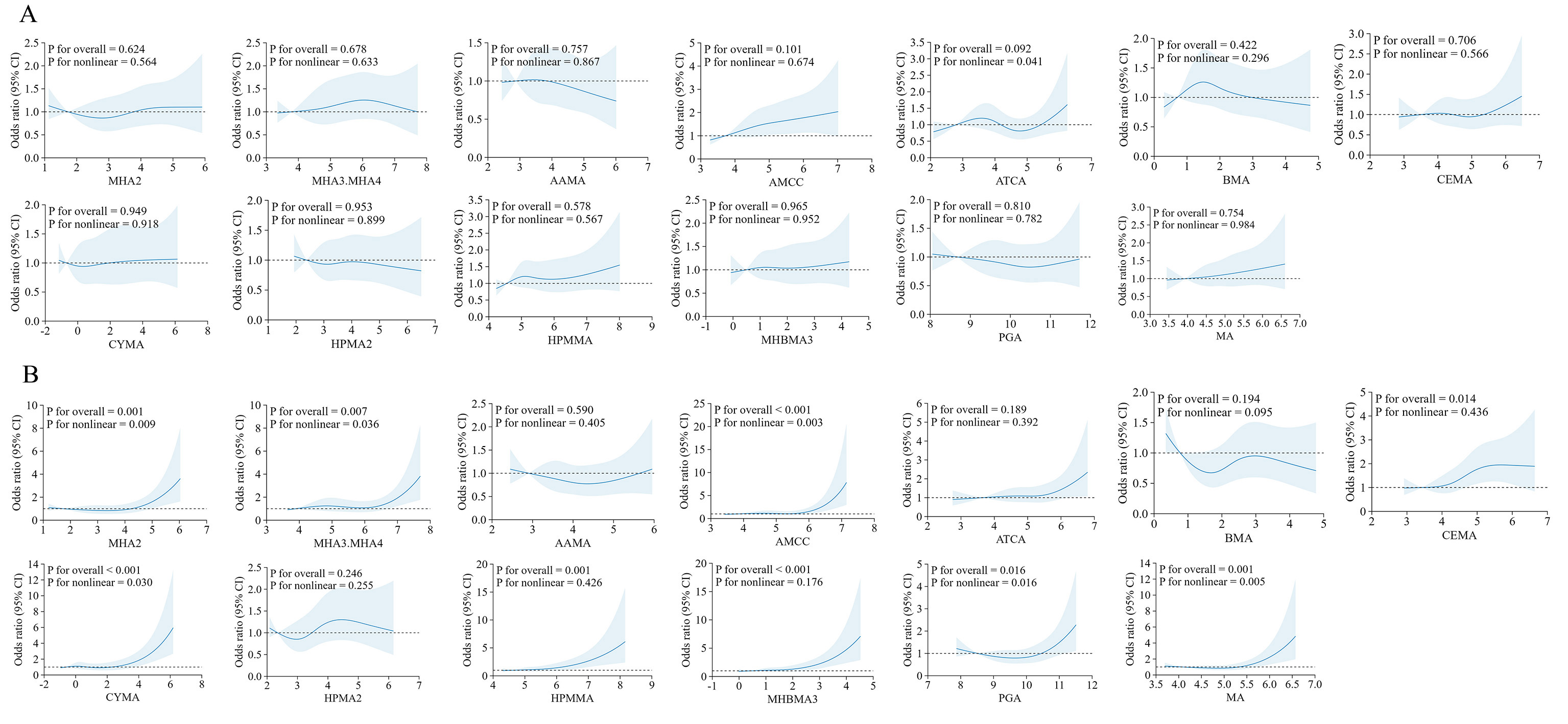


Figure S5 RCS plots of the association between the concentration of urinary VOCs and hyperlipidemia, stratified by sex: (A) Male, (B) Female. The model was adjusted for covariates including sex, age, race/ethnicity, PIR, education level, marital status, BMI, smoking status, alcohol consumption, and urinary creatinine level. RCS, Restricted Cubic Splines; VOCs, volatile organic compounds; PIR, poverty-income ratio; BMI, body mass index.


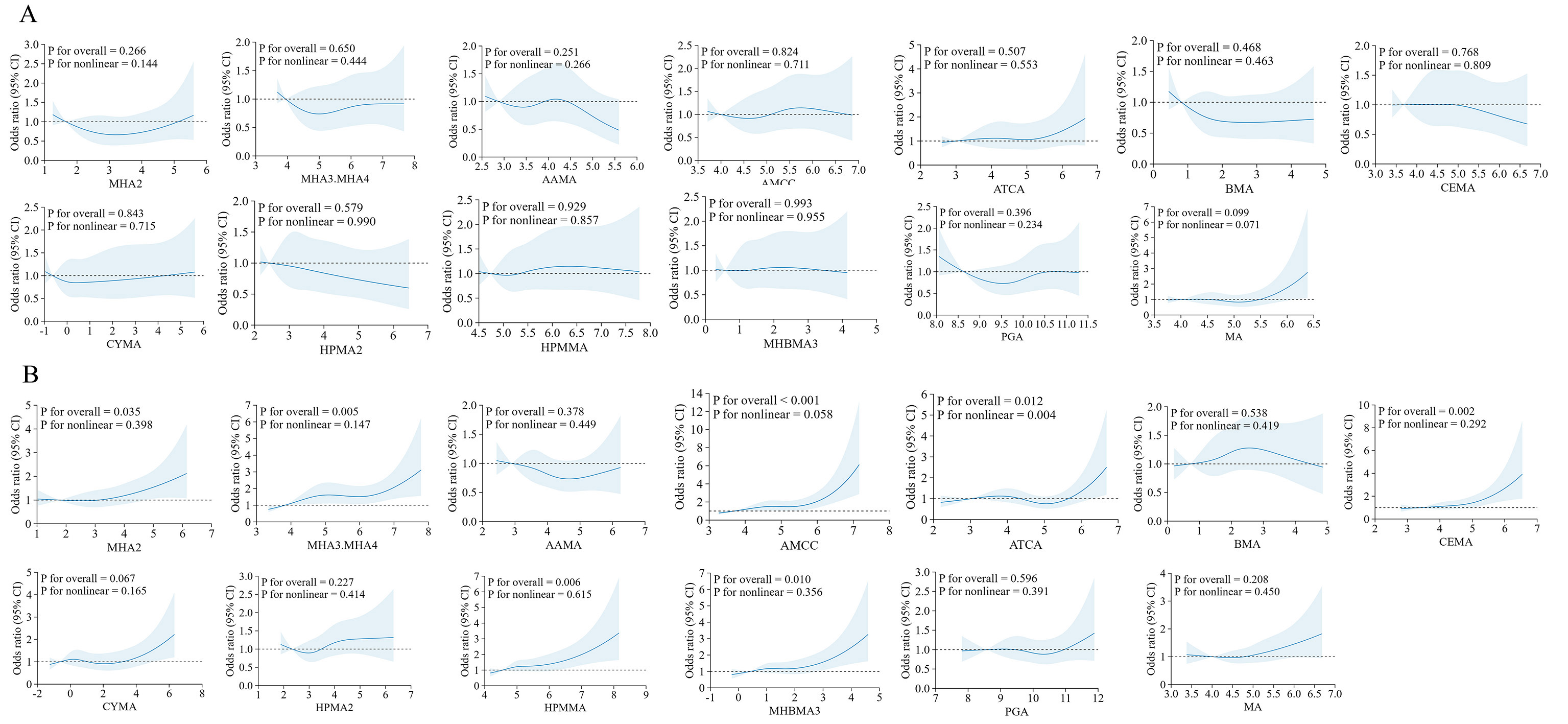


Figure S6 RCS plots of the association between the concentration of urinary VOCs and hyperlipidemia, stratified by age group: (A) Age < 60 years, (B) Age ≥ 60 years. The model was adjusted for covariates including sex, age, race/ethnicity, PIR, education level, marital status, BMI, smoking status, alcohol consumption, and urinary creatinine level. RCS, Restricted Cubic Splines; VOCs, volatile organic compounds; PIR, poverty-income ratio; BMI, body mass index


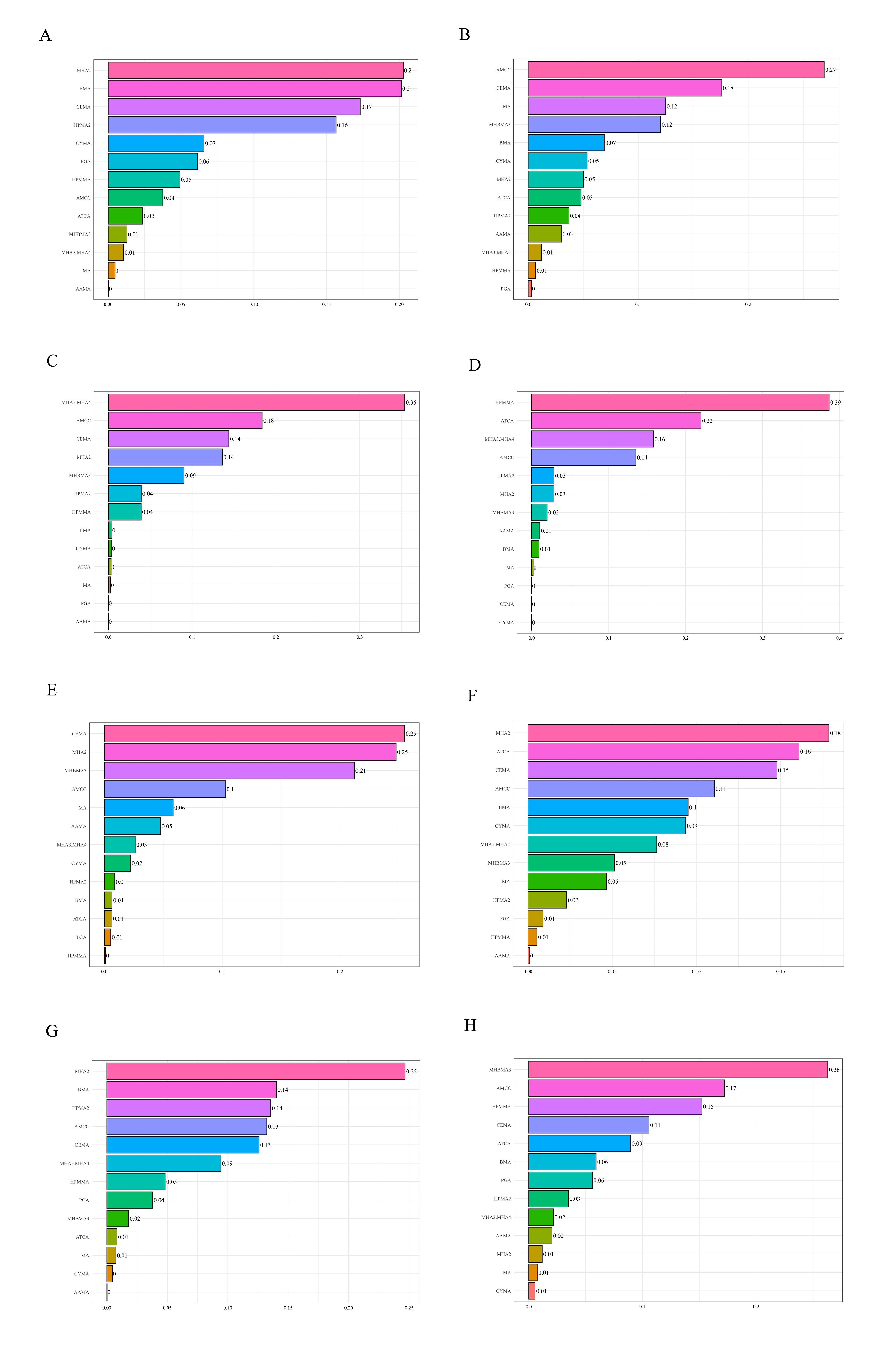


Figure S7 WQS model results for the association between VOCs mixtures (ln-transformed) and hyperlipidemia, stratified by sex and age group. Panels A–D display results from the positive-direction WQS model, and panels E–H display results from the negative-direction WQS model. Panels (A) & (E): males; (B) & (F): females; (C) & (G): age < 60 years; (D) & (H): age ≥ 60 years. The model was adjusted for covariates including sex, age, race/ethnicity, PIR, education level, marital status, BMI, smoking status, alcohol consumption, and urinary creatinine level. WQS, weighted quantile sum; VOCs, volatile organic compounds.


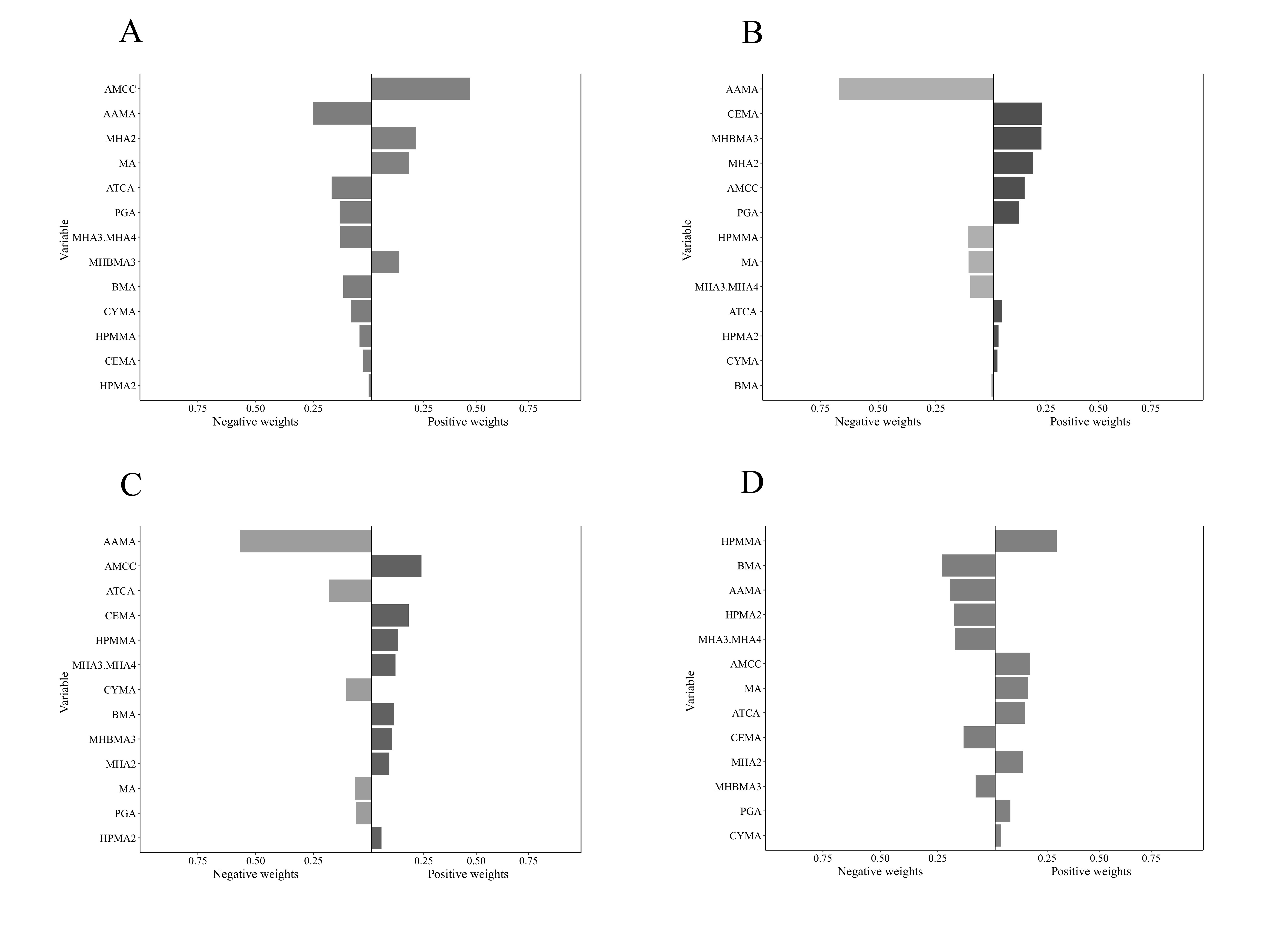


Figure S8 Qgcomp model results for the association between VOCs mixtures and hyperlipidemia, stratified by sex and age group: (A) Male, (B) Female, (C) Age < 60 years, (D) Age ≥ 60 years. The model was adjusted for covariates including sex, age, race/ethnicity, PIR, education level, marital status, BMI, smoking status, alcohol consumption, and urinary creatinine level. Qgcomp, Quantile-based g computation method; VOCs, volatile organic compounds.


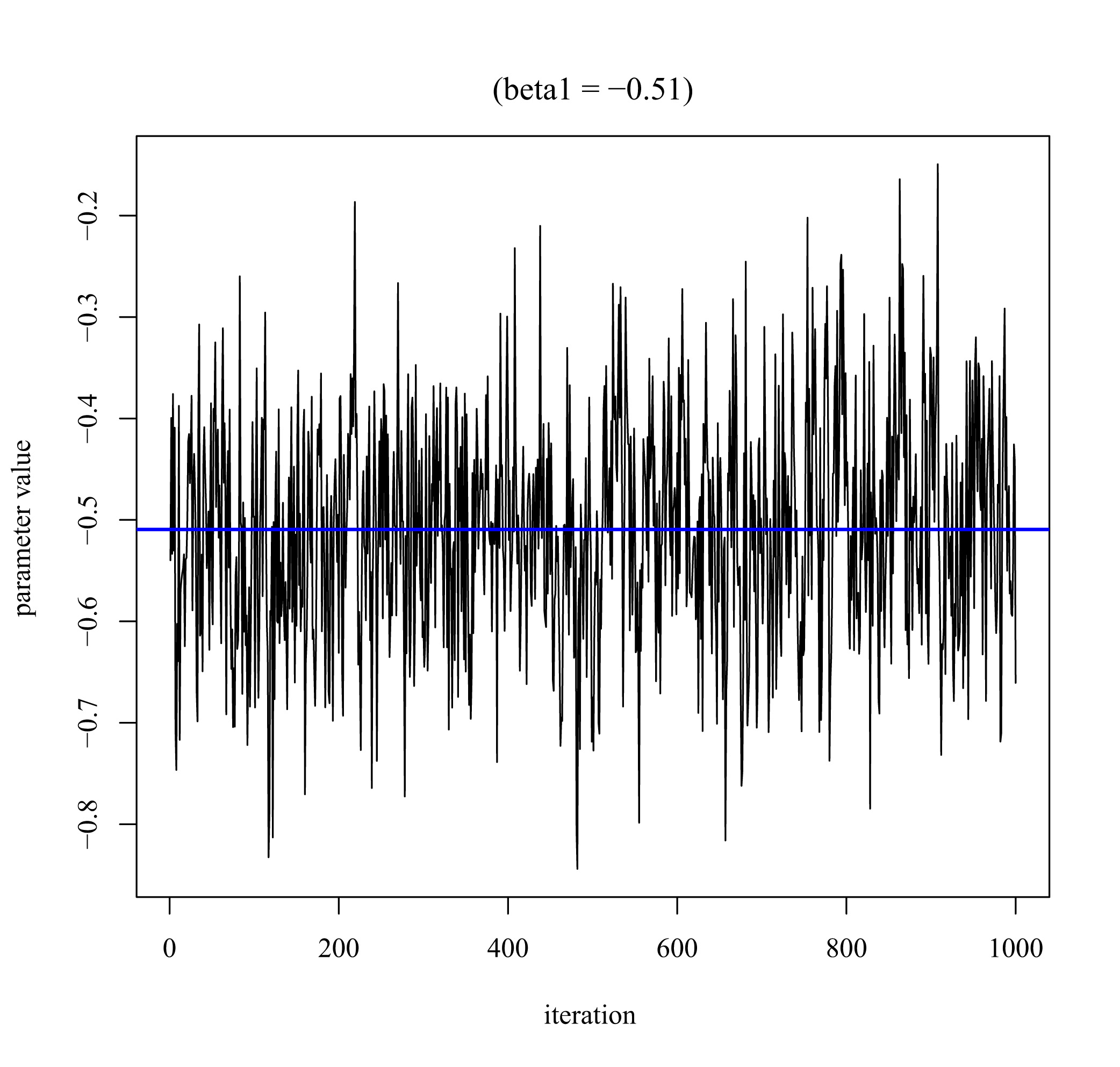


Figure S9 Trace plots of the beta1 parameters during MCMC sampling in the BKMR model.


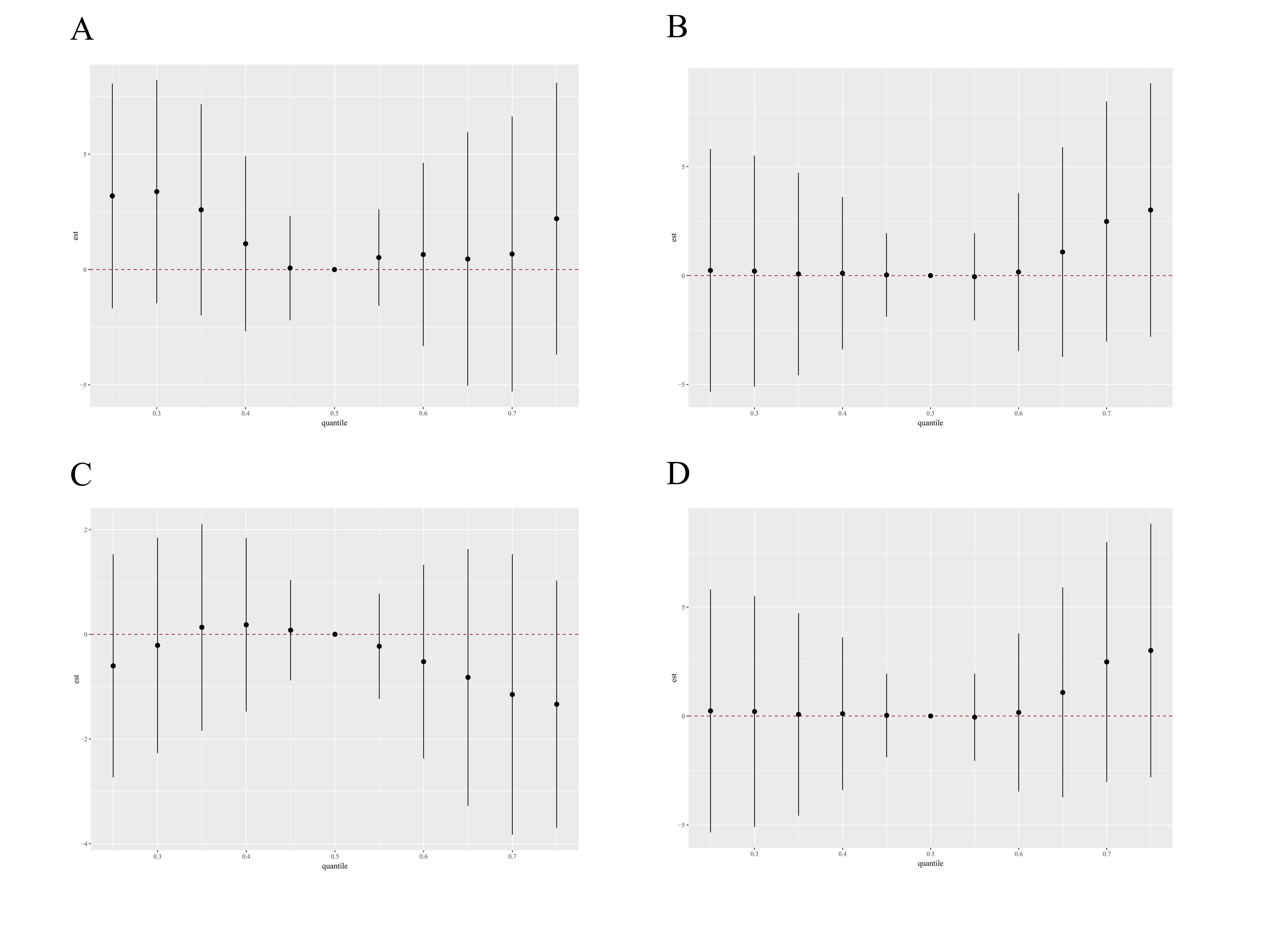


Figure S10 Using the BKMR model, the overall relationship between VOCs mixtures and hyperlipidemia was stratified by sex and age group: (A) Male, (B) Female, (C) Age < 60 years, (D) Age ≥ 60 years. The model was adjusted for covariates including sex, age, race/ethnicity, PIR, education level, marital status, BMI, smoking status, alcohol consumption, and urinary creatinine level. BKMR, Bayesian kernel machine regression; VOCs, volatile organic compounds.


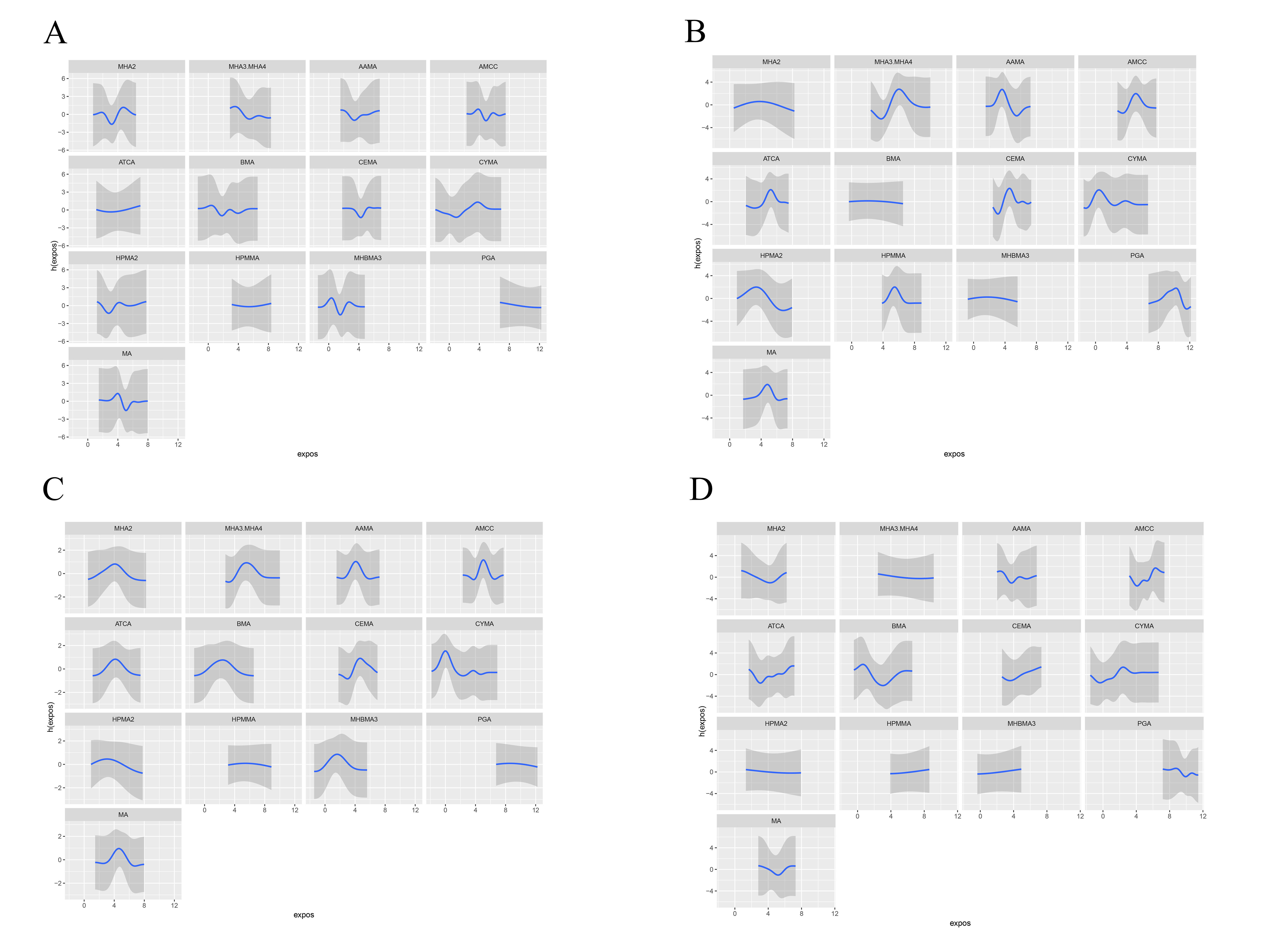


Figure S11 The univariate dose-response relationship between each VOC and hyperlipidemia, evaluated using the BKMR model, with all other VOCs fixed at the 50th percentile, and stratified by sex and age group: (A) Male, (B) Female, (C) Age < 60 years, (D) Age ≥ 60 years. The model was adjusted for covariates including sex, age, race/ethnicity, PIR, education level, marital status, BMI, smoking status, alcohol consumption, and urinary creatinine level. BKMR, Bayesian kernel machine regression; VOCs, volatile organic compounds.


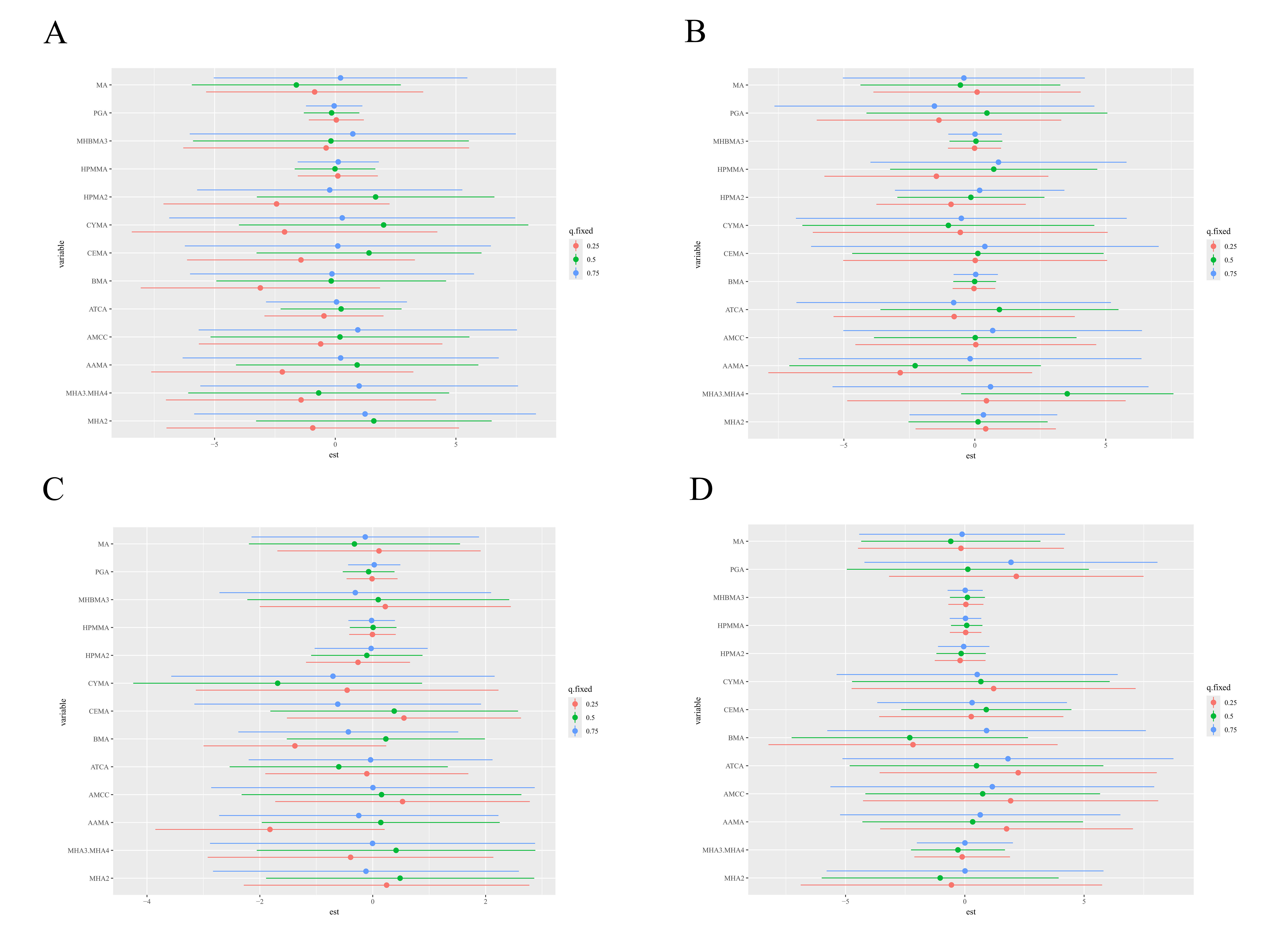


Figure S12 The single-exposure risk of each VOC on hyperlipidemia, evaluated using the BKMR model, with all other VOCs fixed at the 25th, 50th, or 75th percentile, and stratified by sex and age group: (A) Male, (B) Female, (C) Age < 60 years, (D) Age ≥ 60 years. The model was adjusted for covariates including sex, age, race/ethnicity, PIR, education level, marital status, BMI, smoking status, alcohol consumption, and urinary creatinine level. BKMR, Bayesian kernel machine regression; VOCs, volatile organic compounds.





Figure S13 The response of a single VOC, with the second VOC fixed at various percentiles (25th, 50th, 75th), and the remaining VOCs fixed at the 50th percentile, evaluated using the BKMR model and stratified by sex and age group: (A) Male, (B) Female, (C) Age < 60 years, (D) Age ≥ 60 years. The model was adjusted for covariates including sex, age, race/ethnicity, PIR, education level, marital status, BMI, smoking status, alcohol consumption, and urinary creatinine level. BKMR, Bayesian kernel machine regression; VOCs, volatile organic compounds.


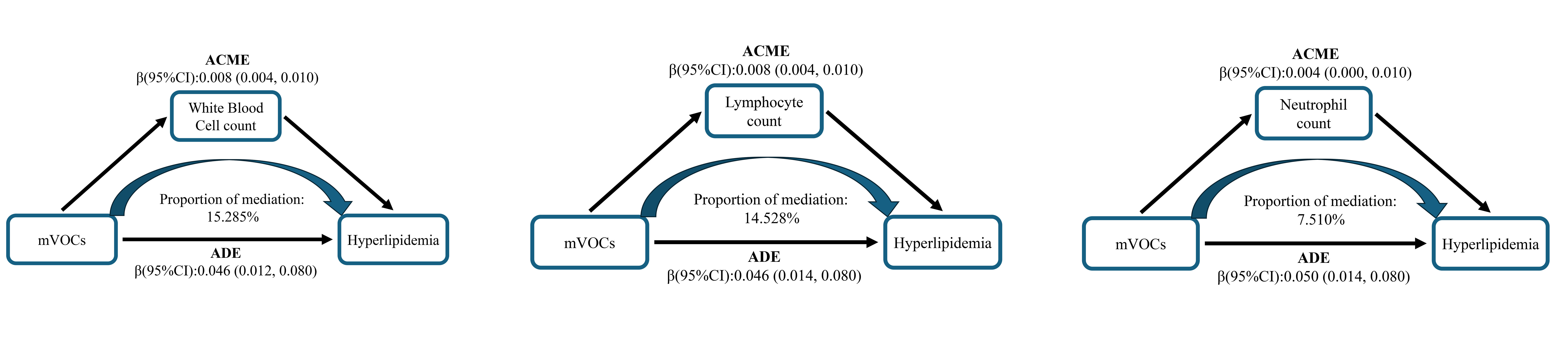


Figure S14 Mediation effects of a single inflammatory marker in the association between urinary VOCs and hyperlipidemia. The model was adjusted for covariates including sex, age, race/ethnicity, PIR, education level, marital status, BMI, smoking status, alcohol consumption, and urinary creatinine level. VOCs, volatile organic compounds; ACME, Average Causal Mediation Effect; ADE, Average Direct Effect.
